# Vertical Distribution and Diversity of Phototrophic Bacteria within a Hot Spring Microbial Mat (Nakabusa Hot Springs, Japan)

**DOI:** 10.1264/jsme2.ME19047

**Published:** 2019-12-27

**Authors:** Joval N. Martinez, Arisa Nishihara, Mads Lichtenberg, Erik Trampe, Shigeru Kawai, Marcus Tank, Michael Kühl, Satoshi Hanada, Vera Thiel

**Affiliations:** 1 Department of Biological Sciences, Graduate School of Science, Tokyo Metropolitan University 1–1 Minami-Osawa, Hachioji, Tokyo 192–0397 Japan; 2 Department of Natural Sciences, College of Arts and Sciences, University of St. La Salle Bacolod City, 6100 Negros Occidental Philippines; 3 Bioproduction Research Institute, National Institute of Advanced Industrial Science and Technology (AIST) Tsukuba, Ibaraki Japan; 4 Marine Biological Section, Department of Biology, University of Copenhagen Strandpromenaden 5, DK-3000 Helsingør Denmark

**Keywords:** photosynthetic bacteria, hot springs, microbial diversity, vertical distribution, 16S rRNA gene amplicon sequences

## Abstract

Phototrophic microbial mats are assemblages of vertically layered microbial populations dominated by photosynthetic microorganisms. In order to elucidate the vertical distribution and diversity of phototrophic microorganisms in a hot spring-associated microbial mat in Nakabusa (Japan), we analyzed the 16S rRNA gene amplicon sequences of the microbial mat separated into five depth horizons, and correlated them with microsensor measurements of O_2_ and spectral scalar irradiance. A stable core community and high diversity of phototrophic organisms dominated by the filamentous anoxygenic phototrophs, *Roseiflexus castenholzii* and *Chloroflexus aggregans* were identified together with the spectral signatures of bacteriochlorophylls (BChls) *a* and *c* absorption in all mat layers. In the upper mat layers, a high abundance of cyanobacteria (*Thermosynechococcus* sp.) correlated with strong spectral signatures of chlorophyll *a* and phycobiliprotein absorption near the surface in a zone of high O_2_ concentrations during the day. Deeper mat layers were dominated by uncultured chemotrophic *Chlorobi* such as the novel putatively sulfate-reducing “*Ca.* Thermonerobacter sp.”, which showed increasing abundance with depth correlating with low O_2_ in these layers enabling anaerobic metabolism. Oxygen tolerance and requirements for the novel phototroph “*Ca.* Chloroanaerofilum sp.” and the uncultured chemotrophic *Armatimonadetes* member type OS-L detected in Nakabusa hot springs, Japan appeared to differ from previously suggested lifestyles for close relatives identified in hot springs in Yellowstone National Park, USA. The present study identified various microenvironmental gradients and niche differentiation enabling the co-existence of diverse chlorophototrophs in metabolically diverse communities in hot springs.

Microbial mats are stratified assemblages of microbes and exopolymers that may develop into thick perennial microbial communities in extreme aquatic environments such as hot springs and hypersaline waters. These communities have a limited microbial diversity due to the extreme environmental conditions, and hot spring microbial mats constitute natural model systems for studying microbial diversity, ecophysiology, and population dynamics (6, 40, 51).

In light-exposed hot springs, microbial mats are dominated by phototrophic bacteria at temperatures between 42–74°C (70), and the microbial community composition and distribution of these mats exhibit niche differentiation along steep vertical gradients of light, water temperatures, and O_2_ concentrations (26, 39, 45, 46). Previous studies have mostly focused on the importance of phototrophic organisms for primary production in the mats (10, 35, 57), and phototrophic bacteria have been shown to play important biogeochemical roles in facilitating oxygen production, sulfide consumption, and carbon fixation, among others (19, 21, 30, 36, 54, 56). Numerous chemotrophic organisms (*e.g.*, sulfate reducers, nitrogen fixers, and H_2_ producers and consumers) co-exist and interact with phototrophs in nutrient cycling within microbial mats (7, 43, 48, 54, 62, 63, 65).

Microbial community compositions in phototrophic hot spring microbial mats have been studied worldwide, including Russia (9, 19, 55), Chile (34), China (30), and the USA (25, 62, 64, 68). Well-developed phototrophic mats characterized by a green upper layer and orange undermat have been studied extensively in the alkaline Octopus Spring and Mushroom Spring in Yellowstone National Park (YNP, USA) (25, 62, 63). Similar mat characteristics have been observed in Nakabusa hot springs in Japan, which have been actively studied for the last few decades (7, 14, 24, 39, 40, 42–44, 46, 48, 59). Mushroom Spring and Nakabusa hot springs are both slightly alkaline (pH 8.0 and ~8.5, respectively) hot springs with similar water chemistries (20, 52). In both hot springs, phototrophic microbial mats with similar characteristics (*e.g.*, a green upper layer over an orange-colored undermat) and communities develop (15, 22). Green mats developing at approx. 60°C in both springs contain oxygenic cyanobacteria, anoxygenic phototrophic *Chloroflexi*, as well as sulfate-reducing, sulfur-oxidizing, and fermenting chemotrophic bacteria (7, 25, 48). Although the community is highly similar at the phylum and class levels and sometimes at the genus level, it generally differs in the inhabiting species.

Microbial mats developing in the slightly alkaline Nakabusa hot springs form various green, brown, orange, and red-colored mats depending on the temperature and biogeochemical conditions in the effluent channels of the springs (15, 39). Previous studies reported the presence of various phototrophic as well as non-phototrophic members in these hot spring mats. The discovery of *Chloroflexus* ( *Cfl.*) *aggregans* and *Roseiflexus* ( *Rof.*) *castenholzii* (13, 14) demonstrated the occurrence of filamentous anoxygenic phototrophic bacteria in green mats developing at 40–65°C, typically in close association with oxygenic cyanobacteria (46). Nakagawa and Fukui (39) initiated a molecular study on microbial community structures in different-colored mats and streamers developing at temperatures of 48–76°C in Nakabusa hot springs and detected markedly different communities below and above 60°C. Microbial mats at lower temperatures were predominated by phototrophic bacteria, while mats at or above 66°C consisted of purely chemotrophic members. Another study on this hot spring found that cyanobacteria were the main component at 52°C up to approximately 60°C (7), while anoxygenic phototrophic bacteria, but not cyanobacteria, were detected in olive-green microbial mats at 65°C in addition to purely chemoautotrophic microbial streamers containing sulfate-reducing members at 75°C (27). Successive increases in the diversity of oxygenic and anoxygenic phototrophic bacteria were found to occur with decreasing temperatures in Nakabusa hot springs (7). In the present study, we selected microbial mats that developed in a small pool in Nakabusa hot springs with temperatures between 56 and 64°C, which contained both oxygenic and anoxygenic phototrophic bacteria, to study the vertical distribution of phototrophs in these mat communities.

The effects of environmental factors, such as temperature and light quality (different wavelengths), on the composition of microbial mat communities have already been investigated in Nakabusa hot spring microbial mats (20, 42, 59); however, the mechanisms by which the vertical distribution of microbial community members changes over seasons as affected by the vertical gradients of light and oxygen at this hot spring remain unknown. To clarify how the vertical distribution of phototrophs and the most abundant chemotrophic microbial community correlate with the microenvironmental gradients of light and oxygen, we examined green microbial mats developing in a small pool in Nakabusa hot springs. In the present study, we used a combination of microsensors to assess environmental parameters (*i.e.*, light quality and oxygen concentrations) and 16S rRNA gene amplicon sequence analyses to identify microbial community members and elucidate their vertical distribution in hot spring phototrophic mats. We also described the diversity and stability of phototrophic microbial mat communities.

## Materials and Methods

### Sample collections and sites

**Fig. 1:**
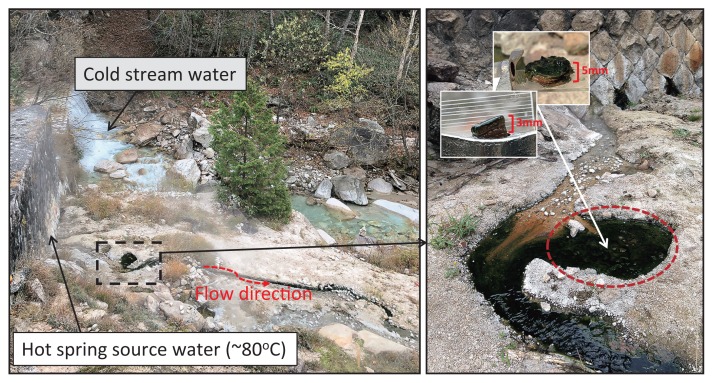
Nakabusa hot springs (Nagano Pref., Japan) “Stream Site=Site B” showing a small pool with green microbial mats (dashed square in the left photo) and enlarged in the right photo. The sampled area is circled in red. Temperatures at the sampling area ranged between 56 and 64°C depending on the sampling time points (Table S1). The inset in the right photo shows representative samples of the mat taken from the sampling point.

Nakabusa hot springs (Nagano Prefecture, Japan) contain two characteristic sampling sites, the ‘Wall Site=Site A’ and ‘Stream Site=Site B’, as described by Nishihara and colleagues (43). In the present study, samples of microbial mats were taken from a small pool of a slightly alkaline hot spring (≈pH 8.9) at ‘Stream Site=Site B’ at six different time points (36°23′33″N, 137°44′52″E; Fig. 1, Table S1) and were characterized by a green upper layer and orange undermat. Water temperature in the pool over the years has varied between 56 and 64°C. In November 2016, two sets of triplicate mat samples were taken, sequenced separately, and averaged, while single samples were taken and analyzed at all other time points. Microbial mat samples (thickness of approx. 3 mm) were taken in June, July, and November 2016 using a #4 cork borer (diameter of 7 mm, used for a bulk community analysis) and another set of mat samples (thickness of approx. 5 mm, separated into individual layers and used in an analysis of vertical distribution) were taken in May and November 2017 using a 4-cm modified cork borer made out of a falcon tube (Table S1). Samples for the bulk community analysis were cut at a depth of 3 mm, while samples taken in May and November 2017 were separated into five different (thickness of approx. 1 mm) layers with a sterile scalpel, and each layer was placed in a separate 2-ml screw cap collection tube. Samples were immediately frozen on dry ice on-site and kept at −80°C in the laboratory for further processing.

### Microsensor measurements of light and oxygen

**Fig. 2:**
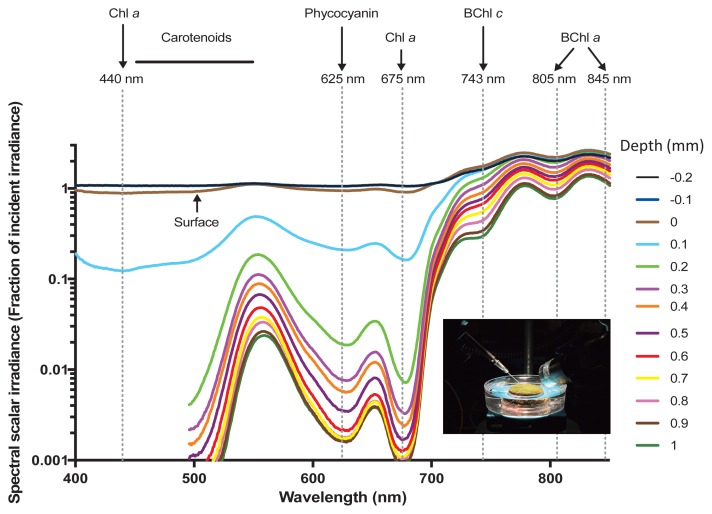
Spectral scalar irradiance measured in different depths in the hot spring microbial mat (inset photo) in November 2016 (Sample GP_56). Absorption maxima of photopigments corresponding to minima/shoulders in the scalar irradiance spectra are indicated with arrows and dashed vertical lines.

Measurements of O_2_ over depth were conducted *in situ* in the microbial mat and overlying spring water in November 2016 at approximately 10:00 AM (Fig. 2) using electrochemical O_2_ microsensors as previously described (18). In order to match the sampling time of the layered samples in November 2017, microsensor data from the same time of day in November 2016 are presented here (10:00 AM) (Fig. 2). O_2_ concentrations were measured using a Clark-type O_2_ sensor (OX25; Unisense A/S, Aarhus, Denmark) with a tip diameter of <25 μm, low stirring sensitivity (<1–2%), and fast response time (T_90_<0.5 s). Microsensors were mounted in a custom-made sensor holder, mounted on a motorized micromanipulator (Unisense A/S), and connected to a PC-interfaced microsensor multimeter (Unisense A/S), both were controlled by dedicated data acquisition and positioning software (SensorTrace Pro; Unisense A/S). Microsensors were carefully positioned at the mat surface (defined as 0 μm) by manual operation of the micromanipulator.

Intact microbial mat samples were brought back to the laboratory for analyses of spectral light penetration using fiber-optic scalar irradiance microsensors (29, 53) according to Nielsen *et al.* (41). The depth profiles of photon scalar irradiance in the mat were measured with fiber-optic scalar irradiance microprobes with a sphere diameter of 80 μm and an isotropic angular response (53). The scalar irradiance microprobe was connected to a fiber-optic spectrometer (USB2000+; Ocean Optics, Florida, USA) interfaced to a PC running spectral acquisition software (Spectra Suite; Ocean Optics). Light was provided vertically from above by a fiber optic tungsten halogen lamp (KL2500-LCD; SCHOTT Benelux B.V., Culemborg, Netherlands) equipped with a collimating lens, while the scalar irradiance microprobe was inserted into the mat at a 45° angle. All measurements were performed in a dark room to avoid stray light. Profiles of photon scalar irradiance were measured in vertical steps of 0.1 mm from 0.2 mm above the surface until no more light was detectable. Incident light was quantified as downwelling photon scalar irradiance from the fiber optic tungsten halogen lamp with the fiber optic microprobe positioned over a black, non-reflective light well at a distance and position in the light field that was similar to the position of the mat surface; in a collimated light field, downwelling irradiance and downwelling scalar irradiance are identical (28). Absolute incident photon irradiance (PAR, 400–700 nm; in μmol photons m^−2^ s^−1^) was measured with a calibrated photon irradiance meter (ULM-500; Heinz Walz GmbH, Effeltrich, Germany) equipped with a spherical sensor (US-SQS/L; Heinz Walz GmbH) positioned in the light well at a distance similar to the position of the mat surface. The acquired spectra (corrected for dark noise) were integrated over the spectral regions of interest, *i.e.*, PAR (400–700 nm), and the integral was related to the absolute incident photon irradiance to obtain the amount of photosynthetic active radiation at each measuring depth expressed as fractions of incident photon scalar irradiance.

### DNA extraction and sequencing

Genomic DNA was isolated from different bulk mat samples as well as separated layers of mats taken from the sampling site. Samples in June and July of 2016 were extracted based on a chloroform-phenol extraction method in combination with the cetyl trimethylammonium bromide (CTAB) method previously described by Nishihara *et al.* (43) (Table S1). Samples from November 2016, May 2017, and November 2017 were processed using the MO BIO PowerBiofilm DNA extraction kit (Qiagen, Hilden, Germany) following the manufacturer’s protocol. Briefly, microbial mat samples (0.11–0.21 g) were centrifuged prior to extraction to remove excess liquid. All centrifugation procedures were performed at 13,000×*g* at room temperature. Homogenization was performed using FastPrep-24 (MP Biomedicals, Irvine, CA, USA) for 1 cycle at 5 m s^−1^ for 30 s. DNA was eluted in 100 μL BF7 solution, purified, and quantified following the protocol of the dsDNA Broadrange (BR) assay (Life Technologies, Grand Island, NY, USA) using a Qubit 3.0 fluorometer (Invitrogen, Carlsbad, CA, USA). Purified DNA (15 ng) was used to amplify the V4 region of 16S rRNA genes using 515F and 806R primers (4). PCR products in duplicate were pooled, purified, and quantified following the dsDNA BR assay using the Qubit 3.0 fluorometer. Purified PCR products (15 ng) were subjected to paired-end sequencing (2×250 nt) using the Illumina Miseq platform (Illumina, San Diego, CA, USA) at FASMAC (Atsugi, Japan).

### Sequence analysis

Sequence analyses were performed as described previously by Nishihara *et al.* (43) with the difference of the clustered Operational Taxonomic Units (OTUs) of the samples taken in 2016, which were originally classified using the Greengenes 13-8 reference database (37), being reclassified together with the 2017 samples using the SILVA database (Silva_128 release in February 2017). Briefly, raw data obtained from Illumina sequencing were quality filtered and analyzed using Quantitative Insights Into Microbial Ecology (QIIME) (ver 1.9.0) (3). The remaining sequences were clustered into OTUs at a ≥97% nucleotide sequence identity level and classified as described above. OTUs were identified using BLAST (Basic Local Alignment Search Tool) in NCBI (https://blast.ncbi.nlm.nih.gov/) as well as by phylogenetic analyses using the ARB software package (33). Phylogenetic trees based on 16S rRNA gene sequences were reconstructed using the Maximum Likelihood method with the GRT Model in the ARB software package (33). The robustness of tree topologies was tested with 100 bootstrap replicates.

Thirty-seven OTUs were selected in the present study based on the mean abundance of ≥0.5% in two sets of triplicated microbial mat samples taken from one of the sampling time points (Nov 2016). The relative abundance of each OTU at the different time points was computed based on the total number of reads after the removal of singletons at each time point (Table S2) (67). The mean abundance and standard deviation (SD) of all OTUs based on the six time points were calculated. Additionally, low abundance OTUs (<0.2%) with the predicted phototrophic lifestyle were included in the analysis. Each of the OTUs was then compared based on their mean abundance and occurrence in all or only some of the six different time points. Core community members were identified as OTUs that occur in all samples and have a minimum relative abundance of >0.003% in each of the sampling time-points. Species richness estimation (Chao1) was assessed using the online program SpadeR (Species-richness Prediction and Diversity Estimation in R) (5). The diversity and evenness of the taxa from six sampling time points were assessed based on the Shannon Index of Diversity (*H’*). Vertical distribution data were processed based on relative abundance as described above. The *t*-test was employed to compare the significance of differences between the mean relative sequence abundance of the vertical distribution of phototrophs and the most abundant chemotrophs between May 2017 and November 2017.

### Nucleotide sequence accession numbers

The nucleotide sequences reported in the present study were deposited in the DDJB/EMBL/NCBI GenBank database with the following accession numbers LC461540–LC461576.

## Results

### Light penetration and O_2_ distribution in the microbial mat

The spectral composition of scalar irradiance markedly changed over the first millimeter (mm) within the mat (Fig. 2). Light attenuation peaks were assigned to the characteristic absorption spectra of the photosynthetic pigments of phototrophs as previously described (8, 46). Wavelengths corresponding to the absorption maxima of photosynthetic pigments, such as Chl *a* (440 and 675 nm), carotenoids (450–550 nm), and phycocyanin (~620 nm), were strongly absorbed in the upper zone and showed increased attenuation from a depth of 0.2 to 0.9 mm. A shoulder in the scalar irradiance spectra also indicated the presence of low amounts of Chl *f* in the upper 0.3 mm of the mat (~710–720 nm) (46). Collectively, these spectral signatures indicated the presence of dense populations of oxygenic phototrophs in the upper layer of the mat. Near infrared radiation (NIR) was less strongly attenuated in this mat layer, and ~20–80% of incident irradiance remained at a depth of 1 mm. The NIR part of the scalar irradiance spectra showed distinct spectral minima corresponding to the absorption maxima of BChl *c* (~743 nm) and BChl *a* (~805 and 845 nm), which became stronger with increases in depths, indicating the increasing abundance of anoxygenic phototrophic bacteria below the uppermost 1 mm of the microbial mat.

**Fig. 3:**
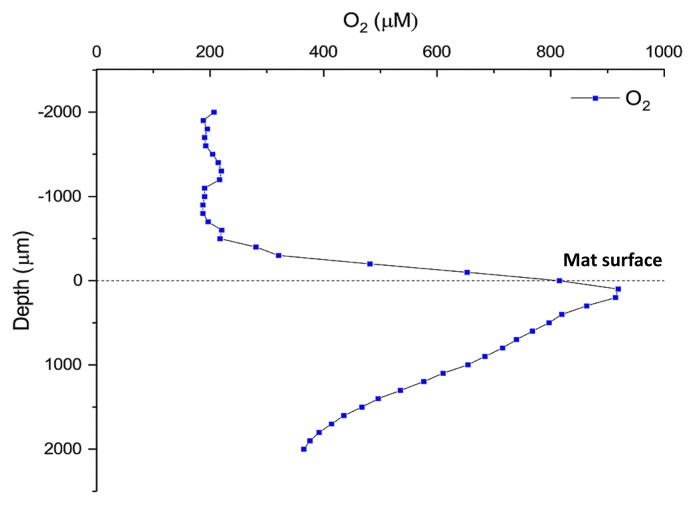
*In situ* microsensor measurements of O_2_ concentrations versus depth in the mat in the Nov. 2016 sampling. The time (10:00 AM) shown in this figure was selected based on the sampling time in November 2017 when mats were sampled for the vertical distribution study.

Oxygen concentrations were measured *in situ* in the upper 2 mm of the mat at a selected measurement time (at 10:00 AM; Fig. 3) during sampling in November 2016. The O_2_ concentration in the overlaying water was ~200 μM, but increased in the diffusive boundary layer above the mat surface and reached a peak concentration of 900 μM 0.2–0.3 mm below the mat surface before steadily decreasing towards deeper mat layers.

### Diversity of phototrophic and most abundant chemotrophic members of the community

The diversity and relative abundance of microbial populations in the phototrophic mats were assessed based on the OTUs of 16S rRNA gene amplicon sequences. High species richness estimates (Chao1, Table 1) were obtained due to the high number of singleton sequences (Table S2), which are generally undetected or rare species that are mostly concentrated on low frequencies (5). Variations in Chao1 estimates between the different time points negatively correlated with the total read numbers, *i.e.*, the sequencing depth. The three time points with high total read numbers (GP_56, GPL_56_M, and GPL_56_N; Table S2), which were averaged from multiple cores and layers, respectively, showed less singleton sequences and lower Chao1 estimates. The lower Chao1 estimates were assumed to be closer to the ‘real’ species richness in the mats. The number of OTUs for each sampling time-point ranged between 452 and 947 with abundance between 2 to 33,510 reads per OTU. Diversity was assessed by the Shannon Diversity Index having a mean value of 4.12 (Table 1), which was similar to previous diversity evaluations (4.71) of the microbial community in Nakabusa hot springs (42). The community was characterized by low evenness (Fig. S1) and was dominated by only a few OTUs in the present study (Table 2), similar to microbial mat communities from Mushroom Spring in YNP (USA) (63).

**Fig. 4:**
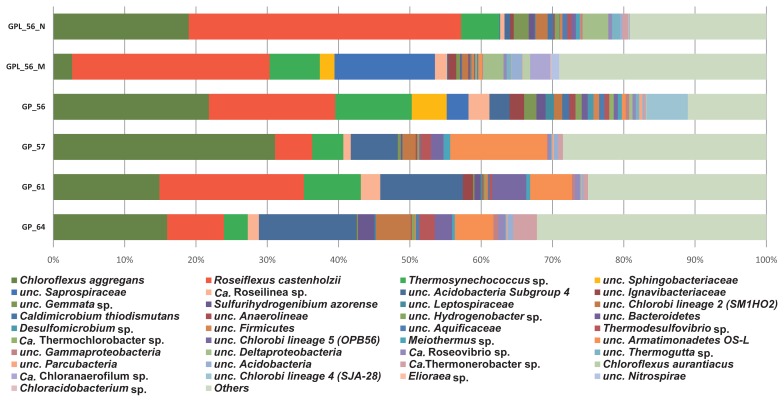
Microbial community based on 16S rRNA gene amplicon sequences at the species level. Thirty-seven selected OTUs, which all showed ≥0.5% mean relative abundance in the two sets of triplicate samples in November 2016, were included for this figure. The identities of the OTUs were based on the SILVA database (Silva_128 released in February 2017) and NCBI database (2018). Sample codes on the left side indicate different sampling time points and temperatures during sampling (GPL_56_N-Nov 2017, 56°C; GPL_56_M-May 2017, 56°C; GP_56-Nov 2016, 56°C; GP_57-July 2016, 57°C; GP_61-June 2016, 61°C; GP_64-July 2016, 64°C).

Among the thirty-seven OTUs (Fig. 4), twenty-five core community members (highlighted in Table 2) that frequently occurred in all samples were identified in the phototrophic mat at a temperature range of 56–64°C. Nine out of the twenty-five OTUs were highly abundant (≥1% mean relative abundance): four were phototrophic bacteria, three were anoxygenic phototrophic bacteria, and one was an oxygenic cyanobacterium. The most abundant OTU, NK_OTU-002 (19.5% mean relative abundance) represents the anoxygenic phototrophic *Chloroflexi* (Fig. S2) member *Rof. castenholzii* with 100% nucleotide sequence identity to the type strain HL08^T^ previously isolated from the Nakabusa mats (14). The second most abundant member, *Cfl. aggregans* NK_OTU-001 (17.6% mean relative abundance) belonging to the same phylum (Fig. S2), was also previously detected in these mats (NKB_63_10) (7) and is 99% identical to *Cfl. aggregans* type strain MD-66^T^ (NCBI Acc. NR_074226) (13). The third most abundant anoxygenic phototrophic filamentous *Chloroflexi* that was frequently detected in all samples, NK_OTU-006, represents a close relative to “*Candidatus (Ca.)* Roseilinea gracile” (62), which was initially detected in Mushroom Spring in YNP in a metagenomic analysis (63, 64) and is currently being examined for isolation and characterization (62). The same organism was found to increase in sequence abundance when subjected to an *in situ* light experiment in Nakabusa hot springs (42), supporting its phototrophic lifestyle. Another abundant phototroph, NK_OTU-003 is 100% identical to *Thermosynechococcus* sp. NK55 (58), which is a known oxygenic phototroph in these mats belonging to *Cyanobacteria* (Fig. S3).

The five other highly abundant core community members, NK_OTU-007, 028, 012, 023, and 051, are predicted to be chemotrophic members of the community, representing three bacterial phyla (Table 2). They are uncultured species and only have 85–96% similarities to the nearest type strains. Among this group, NK_OTU-007 represents the phylum *Acidobacteria* (Fig. S4) and is the most abundant chemotrophic member in this community (5.9% mean relative abundance). It is identical to OTU ‘denovo3451’ (100% nucleotide sequence similarity) previously detected in Nakabusa hot springs (43) and 96% identical to the *Acidobacteria* clone YNP_SBC_BP4_B26 from YNP (38). The phylum *Armatimonadetes* (Fig. S5) is represented by NK_OTU-028 in the present study, which is also higher in abundance than other chemotrophs (4.3% mean relative abundance). This member is 100% identical to an uncultured clone sequence obtained from Nakabusa at 56°C (7) and closely related to clone MS-B_OTU-03 from Mushroom Spring (63) and uncultured *Eubacterium* sp. OS-L from Octopus Spring (69) with 98 and 96% nucleotide sequence similarities, respectively. The results of a partial genome analysis indicated aerobic or microaerobic metabolism for the YNP mat member (63).

Three abundant core community members representing the super phylum *Chlorobi/Bacteroidetes/Ignavibacteria* (Fig. S6) were detected in these mats (OTUs NK_OTU-12, −23, and −51, Table 2). NK_OTU-012 belongs to ‘*Chlorobi* lineage 2’ (Fig. S6) and has 100% sequence identity with the NKB_56_U2 clone previously recovered from Nakabusa hot springs (7) and is 99% identical to *Chlorobi* clone SM1H02, which was initially detected in the Mammoth hot spring, YNP (NCBI Acc. AF445702). NK_OTU-023 represents an uncultured member of ‘*Chlorobi* lineage 5’ (also known as “clade OPB56” and/or “*Ca.* Kapabacteria”, Fig. S6), which is 100% identical to the clone MS-B_OTU-24 detected from Mushroom Spring in 2012 (63). Another ‘*Chlorobi* lineage 5’ member, NK_OTU-51 (Fig. S6), which is 99% similar to the clone MS-B_OTU-29 recovered from Mushroom Spring, YNP, is predicted to represent the newly discovered putative sulfate-reducing bacterium “*Ca.* Thermonerobacter thiotrophicus” (63, 66).

Other core members of the microbial community in Nakabusa hot springs are frequently occurring in all samples, but are less abundant (ranging between 0.2 and 0.9% mean relative abundance). Among the sixteen OTUs detected, only one member, the *Alphaproteobacteria* member NK_OTU-032 (Fig. S7), was related to a strain with predicted phototrophic ability, while all others were putative chemotrophic species (Table 2). NK_OTU-032 is 98% identical to “*Ca.* Roseovibrio tepidum” strain MS-P3, which was initially detected in Mushroom Spring (YNP) in 2015 and is a novel BChl-*a* containing α-proteobacterial species (62). This strain shows 16S rRNA gene sequence similarities to the *Roseomonas* and *Rhodavarius* spp. strains. However, in contrast to non-phototrophic *Roseomonas* species, such as *R. estuarii*, this is the only member of this group that produces BChl *a* and contains *pufLM* genes (encoding for the L and M subunits of the type-2 photosynthetic reaction center) (63), suggesting a chlorophototrophic lifestyle.

Twelve OTUs representing six phyla were only detected at a relative abundance of <0.003% at some time points (Table 2). Five of these OTUs were classified as phototrophs based on their closest relative belonging to four phyla. The phylum *Chloroflexi* (Fig. S2) is represented by two filamentous anox-ygenic chlorophototrophs, *Chloroflexus* sp. NK_OTU-092, which corresponds to *Cfl. aurantiacus* J-10-fl^T^ (NCBI Acc. NR_074263) (49), and NK_OTU-222 with 98% sequence similarity to the *Oscillochloris*-like chlorophototroph “*Ca.* Chloranaerofilum corporosum”, a novel BChl *c* containing filamentous anoxygenic phototrophic member of *Chloroflexi* initially detected in Mushroom Spring, YNP (62). The latter was exclusively detected in the undermat of Mushroom Spring and expected to prefer the anaerobic environment of the deeper layers of the mat (63). NK_OTU-021 may represent an unusual phototrophic member of the phylum *Chlorobi*, “*Ca.* Thermochlorobacter aerophilum”, the first aerobic, photoheterotrophic *Chlorobi* that was initially detected in Octopus Spring, YNP (32, 62). An anoxygenic phototrophic proteobacterium, represented by NK_OTU-279 in the present study represents a member of the genus *Elioraea* (Fig. S7), with 100 and 94% nucleotide sequence similarities to *Elioraea tepidiphila* TU-7^T^ (1) and “*Ca.* Elioraea thermophila”, respectively (Table 2). Although the type strain has been described as a chemotroph, members of this genus are hypothesized to be aerobic anoxygenic phototrophs based on the presence of photosynthesis-related genes in their genomes as well as their ability to synthesize BChl *a* under aerobic conditions (62). Similar to the low-abundance member found in the mat community at 60°C in Mushroom Spring (63), *Elioraea* sp. NK_OTU-279 was also found to be low in abundance in Nakabusa hot springs in the present study. Another low-abundance, less frequently occurring OTU, NK_OTU-15047, putatively represents a novel species of the phototrophic *Acidobacteria* genus *Chloracidobacterium* ( *Cab.*) (97% nucleotide sequence similarity to *Cab. thermophilum* strain B^T^) (2, 11, 60, 61).

We only found few sequences (<100 sequence reads) that were related to *Leptolyngbia* sp. Nb3F1 (Fig. S3) in the present study. *Leptolyngbia*-like NK_OTU-090 was only 91% similar to the Chl-*f* producing *Leptolyngbia* spp. detected in a previous study (46); however, this may indicate another species of Chl-*f* producing cyanobacteria in the microbial mats at Nakabusa hot springs.

### Vertical distribution of phototrophic community members

**Fig. 5:**
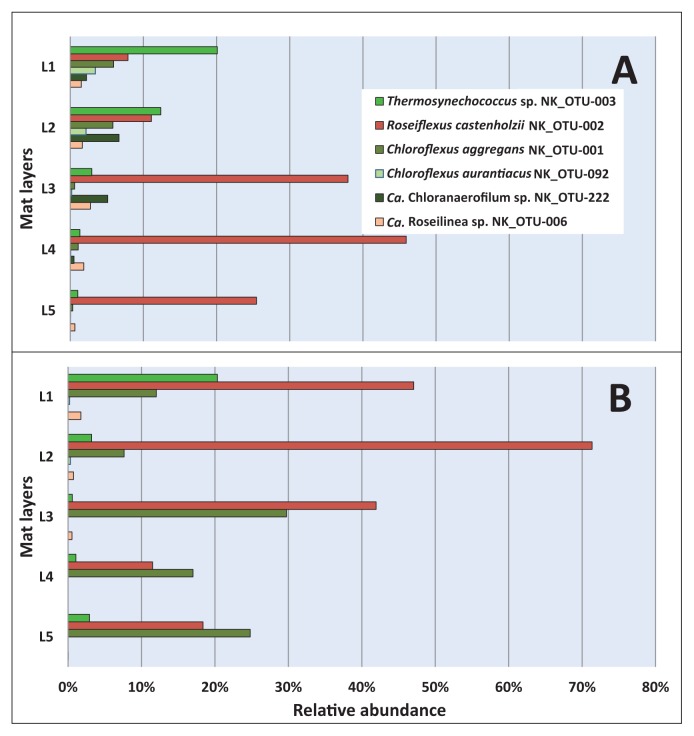
Vertical distribution of phototrophic members of *Chloroflexi* and *Cyanobacteria* based on 16S rRNA gene amplicon sequences in May 2017 (A) and November 2017 (B). Mat layers are indicated by L1 to L5 (L1-uppermost layer; L5-bottom layer).

At two of the time points (May 2017 and November 2017), the mat was separated into five layers that were individually analyzed by 16S rRNA gene amplicon sequencing, disclosing the vertical distribution of different phototrophic and chemotrophic mat members. The sequences of the phototrophic members varied with depth, as shown in Fig. 5. Sequences representing the oxygenic phototroph *Thermosynechococcus* sp., (NK_OTU-003) were similarly abundant (*P*>0.05, Table S3) at both time points and showed a clear decrease in relative abundance with depth (Fig. 5). Sequences representing this cyanobacterium were mainly found in layers 1 and 2, representing the first 2 mm of the mat and correlating with the green color of the upper mat layer as well as strong absorbance at 675 nm (indicating the presence of Chl *a*) in the first 1 mm (Fig. 2). Relative abundance in the second layer was higher in the May sample, correlating with longer and stronger sunlight periods in summer.

Among the anoxygenic phototrophs, sequences representing the most abundant member of the phylum *Chloroflexi*, BChl *a*-containing *Rof. castenholzii* (NK_OTU-002) did not significantly differ (*P*>0.05, Table S3) between the two sampling periods (May and November 2017). Sequences markedly varied among layers (Fig. 5), with the highest relative abundance of *Rof. castenholzii* being observed in layer 4 in May 2017 (Fig. 5A) and layer 2 in the winter sample (November 2017) (Fig. 5B). The higher abundance of BChl *a*-containing *Rof. castenholzii* sequences in deeper layers correlated with the relatively higher light penetration of NIR into deeper layers, particularly in the summer (Fig. 2 and 5A). Differences in the layer with the highest relative sequence abundance for this organism between the two sampling times correlated with the different light conditions between the two seasons. The higher competitiveness of *Rof. castenholzii* in the lower layers may be explained by the avoidance of competition with other phototrophic bacteria, *e.g.*, cyanobacteria and other FAPs; or the active avoidance of high light conditions in the upper layers. The abundance of sequences representing the oxygen-tolerant filamentous anoxygenic phototroph, *Cfl. aggregans* (NK_OTU-001), significantly differed (*P*<0.05, Table S3) between the two seasons, with higher abundance in winter (Nov 2017, 18.35% mean relative abundance) than in summer (May 2017, 2.75% mean relative abundance). Within the mats, *Cfl. aggregans* sequences clearly showed decreasing relative abundance with depth in May 2017 (Fig. 5A), with relatively high abundance in the first two upper layers of the mat. In contrast, in November 2017 (Fig. 5B), overall abundance was markedly higher, indicating the stronger competitiveness of this member in the winter than in the summer months. This correlates with the presence of BChl *c*-containing light-harvesting organelles, the chlorosomes, in this organism, which are often found in low-light-adapted phototrophs such as green sulfur bacteria (47). Another phototrophic member, “*Ca.* Roseilinea sp.” NK_OTU-006 also showed significant differences (*P*=0.05, Table S3) in average abundance between the two sampling seasons, with higher relative abundance in May than in November 2017. This member does not contain BChl *c* or chlorosomes and relies entirely on BChl *a* for its phototrophic growth (62), and, thus, is not as low-light adapted as *Chloroflexus* spp. Furthermore, the vertical distribution of “*Ca.* Roseilinea sp.” NK_OTU-006 differed between the two samplings. The relative abundance of sequences was the highest in layer 3 in May 2017 (Fig. 5A), and in the uppermost layer in November 2017 (Fig. 5B), indicating that irradiance supporting this phototroph penetrated less deeply into the mats in winter. Alternatively, oxygen concentrations were hypothesized to be less favorable (*e.g.*, too high) in the upper layers in May or too low in November in the deeper layers because this organism has been suggested to be an oxygen-tolerant or -dependent anoxygenic phototroph, possibly with the need for microoxic conditions, as has been shown for another phototrophic mat member, *Cab. thermophilum* (61). The two less abundant members of the phylum *Chloroflexi*, *Cfl. aurantiacus* (NK_OTU-092) and “*Ca.* Chloranaerofilum sp.” (NK_OTU-222), showed no significant differences (*P*>0.05, Table S3) between seasons (May 2017 and Nov 2017). However, the sequence abundance of these members among the five layers varied with depth. Anoxygenic filamentous phototrophic *Cfl. aurantiacus* sequences were relatively high in the upper two layers for both seasons, indicating requirements for light and/or oxygen. On the other hand, sequences representing “*Ca.* Chloranaerofilum sp.” (NK_OTU-222), showed the highest relative abundance in the second layer for both sampling seasons, suggesting a preference for lower light and/or oxygen concentrations than *Cfl. aurantiacus* (Fig. 2 and 3). However, in contrast to previous studies on “*Ca.* Chloranaerofilum corporosum” in hot spring mats in YNP, the member of this microbial mat community in Nakabusa did not appear to prefer completely anoxic conditions, as suggested previously (62).

**Fig. 6:**
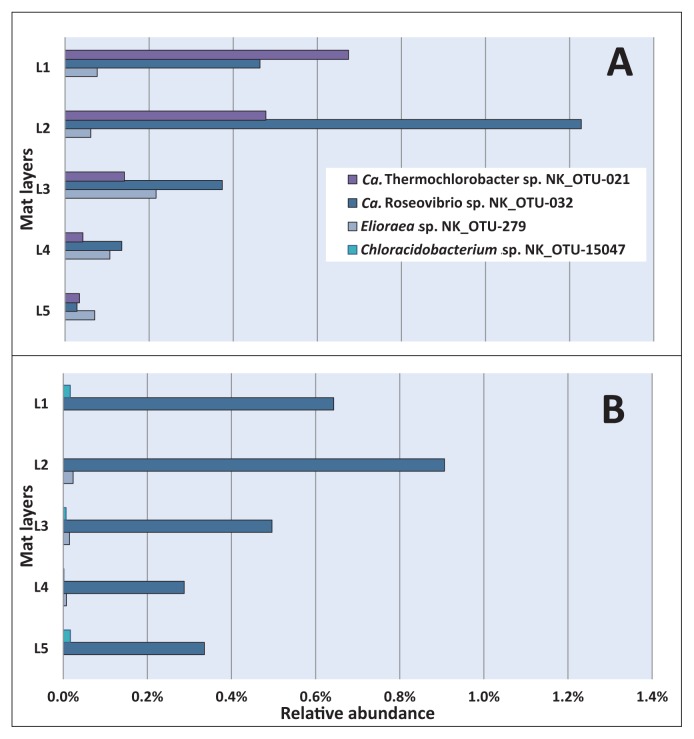
Vertical distribution of less abundant phototrophic members of *Proteobacteria*, *Chlorobi*, and *Acidobacteria* based on 16S rRNA gene amplicon sequences in May 2017 (A) and November 2017 (B). Mat layers are indicated by L1 to L5 (L1-uppermost layer; L5-bottom layer).

The vertical distributions of less abundant phototrophic members represented by three phyla (*Chlorobi*, *Acidobacteria*, and *Proteobacteria*) (Fig. S4, S6, and S7) were also analyzed based on the relative abundance of sequences in the five layers (Fig. 6). “*Ca.* Thermochlorobacter sp.” NK_OTU-021 (*Chlorobi*) was only detected in May 2017 (Fig. 6A), with the highest relative abundance (0.67%) being observed in the uppermost layer and markedly decreasing with depth, correlating with the suggested need and tolerance for high oxygen concentrations for its next relative “*Ca.* Thermochlorobacter aerophilum” (32). Another less abundant member in these mats, *Chloracidobacterium* sp. NK_OTU-15047, was only detected in November 2017 (Fig. 6B) with unexpectedly varying abundance (ranging between 0.01 and 0.02%) from the upper to the lower layer despite its known microaerophilic lifestyle (61). Two less abundant phototrophic members in these mats represent the phylum *Proteobacteria*, “*Ca.* Roseovibrio sp.” NK_OTU-032 and *Elioraea* sp. NK_OTU-279. “*Ca.* Roseovibrio sp.” NK_OTU-032 showed no significant differences (*P*>0.05, Table S3) in the average relative abundances of sequences for the two sampling periods. In a comparison of the relative abundance in five layers, this member showed higher abundance in the second layer representing 1.23 and 0.91% of mean relative sequence abundance in May 2017 and November 2017 (Fig. 6), respectively. A decrease in the relative abundance of sequences was detected after the second layer for both seasons, suggesting the preference of (micro)aerobic or (low-)light conditions. The other proteobacterial member, *Elioraea* sp. NK_OTU-279, representing a putative aerobic anoxygenic phototroph, showed significant differences (*P*<0.05, Table S3) in the averaged relative abundance of sequences between sampling seasons with higher abundance in May 2017 than in November 2017 (Fig. 6). Despite this difference, the vertical distribution of this OTU demonstrated that the organism showed the highest relative abundance in the middle layer in both sampling seasons (layer 3), which correlated with its assumed microaerophilic physiology (62).

### Vertical distribution of the most abundant chemotrophic community members

**Fig. 7:**
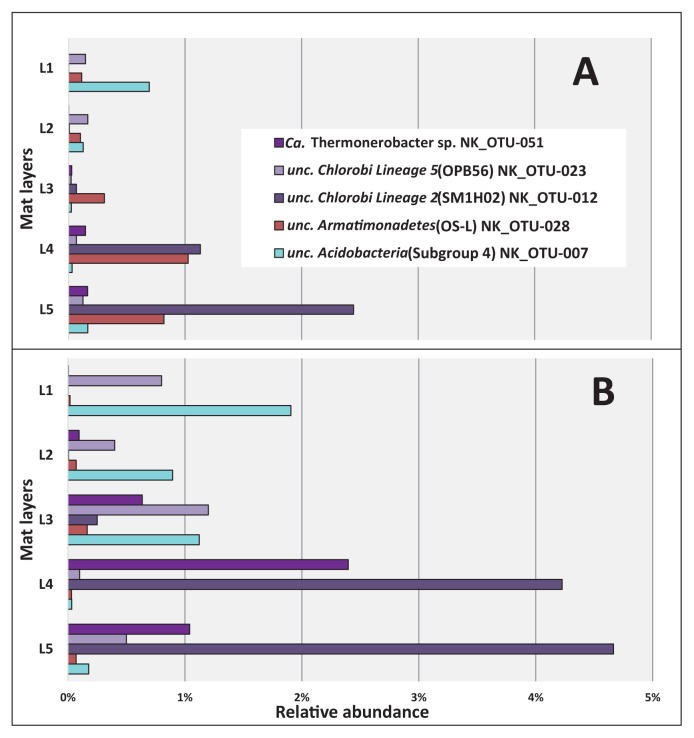
Vertical distribution of the most abundant (≥1%) chemotrophic members of *Chlorobi*, *Armatimonadetes*, and *Acidobacteria* based on 16S rRNA gene amplicon sequences in May 2017 (A) and November 2017 (B). Mat layers are indicated by L1 to L5 (L1-uppermost layer; L5-bottom layer).

The distribution of abundant chemotrophic community members was also analyzed based on the relative abundance of sequences among the five separated layers of these mats (Fig. 7). As shown in Table 2, five out of the nine most abundant members of these community members corresponded to uncultured, putatively chemotrophic species (NK_OTU-007, 012, 023, 028, and 051). Three members, NK_OTU-012, OTU-023, and OTU-051, representing the phylum *Chlorobi* (Fig. S6), were all related to sequences previously detected in hot spring environments in YNP and predicted to have chemoheterotrophic lifestyles (16, 17, 23, 63, 66). The relative abundance of sequences did not significantly differ (*P*>0.05, Table S4) between May 2017 and November 2017, suggesting that these core members were relatively stable in abundance over time. The vertical distribution of the uncultured ‘*Chlorobi* lineage 2’ (SM1H02) represented by NK_OTU-012 and NK_OTU-51 related to “*Ca.* Thermonerobacter thiotrophicus” (66) in the ‘OPB56 clade’ (Chlorobi lineage 5, “*Ca.* Kapabacteria”) showed that the relative abundance of these sequences increased with depth, reflecting higher abundance in the anaerobic zones of the mats (Fig. 3 and 7) and indicating a putatively anaerobic lifestyle. Another ‘*Chlorobi* lineage 5’ (OPB56 clade, “*Ca.* Kapabacteria”) member, NK_OTU-023, showed the highest relative sequence abundance in the second and third layers in May 2017 (0.17% mean relative abundance) and November 2017 (1.2% mean relative abundance), respectively, which indicates a preference for microaerobic environments (Fig. 7).

Other abundant putatively chemotrophic members representing two phyla (*Acidobacteria* and *Armatimonadetes*) (Fig. S4 and S5) also did not significantly differ (*P*>0.05, Table S4) in relative sequence abundance for the two sampling seasons. However, the vertical distribution of *Acidobacteria* member NK_OTU-007 showed the highest relative abundance of sequences in the uppermost layer of the mats, in which cyanobacteria were present and high oxygen concentrations occurred during the day (Fig. 3) and decreased with depth (Fig. 7), indicating a possible aerobic metabolism in both seasons. In contrast to previous studies, sequences representing the uncultured OS-L like *Armatimonadetes* member NK_ OTU-028 showed high relative abundance in the lower layers, specifically in the fourth and fifth layers in May and November 2017, respectively (Fig. 7), indicating putatively anaerobic metabolism.

## Discussion

The diversity, relative abundance, and depth-dependent distribution of the hot spring-associated phototrophic microbial community at Nakabusa hot springs “Stream site=Site B” were revealed in the present study based on 16S rRNA gene amplicon sequencing. This method is suitable for identifying different community members and estimating their relative abundance through sequence reads, but does not disclose their metabolic activity or physiological capacity or ability. The mean diversity index obtained in the present study was similar to that reported previously in Nakabusa hot springs (42) and did not markedly change among the six sampling time points, indicating a stable microbial mat community, which is uneven and dominated by only a few community members (Table 1 and Fig. S1). The core community consists of nine abundant and sixteen less abundant members present in all the samples analyzed (Table 2). The characteristic layering of the green top layer and orange-colored undermat observed in the mats in the present study were also found in phototrophic microbial mats in many hot springs worldwide (9, 22, 34, 50, 55, 63, 64, 72). The green upper layer correlated with the presence of oxygenic cyanobacteria in the upper 1 to 2 mm of the mat, with their photosynthetic pigments giving the layer its characteristic color, while the orange-colored undermat only contained anoxygenic phototrophic and chemotrophic members (Fig. 5–7).

Microbial mats from Nakabusa hot springs were dominated by phototrophic species (Fig. 4 and Table 2), with additional chemotrophic organisms co-existing. Overall, ten different phototrophic bacteria were detected in these mats. Consistent with previous findings, the oxygenic cyanobacteria, *Thermosynechococcus* sp. (58) and filamentous anoxygenic phototrophs (FAPs), *Cfl. aggregans* (13) and *Rof. castenholzii* (14) were identified as the most abundant phototrophic members in the Nakabusa mats in this study (7, 27, 39, 48). Less abundant previously described phototrophs, such as *Cfl. aurantiacus* (12) and *Chloracidobacterium* sp., were also detected. Additionally, some unusual and novel phototrophic members from three phyla (*Elioraea* sp. and “*Ca.* Roseovibrio sp.”, *Proteobacteria*; “*Ca.* Thermochlorobacter sp.”, *Chlorobi*; “*Ca.* Roseilinea sp.” and “*Ca.* Chloranaerofilum sp.”, *Chloroflexi*) were identified in Nakabusa hot springs for the first time, while closely related species have been detected in Mushroom Spring, suggesting their more universal distribution in hot spring environments (62, 65). The presence of some of these phototrophs in Nakabusa hot springs was previously reported by cultivation and microscopy studies (*e.g.*, *Chloracidobacterium* sp. and “*Ca.* Thermochlorobacter sp.”; Y. Shirotori and M. Tank, unpublished data). This illustrates the importance of the combination of traditional and modern techniques in the discovery and identification of unusual, novel, and low-abundance members in these hot spring mats.

Nine members from five bacterial phyla and dominated by the phylum *Chloroflexi* (Fig. S2), including four phototrophs were consistently found in high abundance in these mats (Fig. 4). Similar to the undermat community in Mushroom Spring mats in YNP, *Roseiflexus* spp. was identified as the most abundant member (63), while in contrast to the YNP study, *Cfl. aggregans* co-existed as the second most abundant member in Nakabusa (Table 2). Both FAPs have been detected in these mats before and their phototrophic metabolism in the mats has been supported by *in situ* experiments (42). In addition to ribosomal gene sequences, the presence of these FAPs was demonstrated in the present study by their characteristic photosynthetic pigments BChl *a* and *c* based on the absorption peaks at 743 and 845 nm in the first upper millimeter (Fig. 2). The different photosynthetic pigments allow the two phototrophic *Chloroflexi* members to inhabit different ecological niches and depths in these mats (Fig. 5, and 6). Differences in abundance and depth distribution for these FAPs in the mats between the summer and winter samples may be due to the different light intensities in these seasons. For example, during the high-light season (summer), NIR light penetrates deeper into the mats, allowing the dominating red FAP *Rof. castenholzii* to grow phototrophically in the lower layers of the mat, while they were found closer to the surface in the low-light season (winter) (Fig. 5). In addition to light, oxygen also plays an important role as the determining factor for the vertical distribution of these FAPs (and other mat community members) (Fig. 3). Both species are known to grow phototrophically in the absence of oxygen only, while both have been shown to be additionally able to grow chemotrophically under aerobic dark conditions (13–15). The lower abundance of oxygenic phototrophic cyanobacteria in the mats in winter may be assumed to lead to lower oxygen concentrations, particularly in the intermediate and deeper layers of the mat, thereby facilitating the anoxygenic phototrophic growth of the FAPs. In addition, the lightharvesting apparatus, the so-called chlorosomes, allow *Cfl. aggregans* to efficiently grow under low-light conditions, possibly leading to the higher relative abundance observed in these mats in winter (Fig. 5). Thus, a combination of several factors, including oxygen and light, is hypothesized to shape the environmental conditions leading to the niche separation, vertical distribution, and competitiveness of the different mat members (Fig. 2 and 3). The closely related species *Cfl. aurantiacus*, which is inconsistently present and at markedly lower numbers in Nakabusa spring mats, shows very similar metabolism and growth physiology to *Cfl. aggregans*. Its presence in the upper layers of the mat in the summer sample (Fig. 5A) confirms the described oxygen tolerance for this species and facultative aerobic chemoheterotrophic metabolism (49).

“*Ca.* Roseilinea gracile”, an unusual and novel *Anaerolineae-*like *Chloroflexi* member and predicted red FAP containing BChl *a* was initially detected in YNP (31, 62–64). A recent genomic study placed this organism in the proposed new class “*Ca.* Thermofonsia”, a sister class of *Anaerolineae* from which it may be distinguished particularly by their predicted aerobic metabolism (71) (Fig. S2). Although not as abundant as *Rof. castenholzii*, sequences representing “*Ca.* Roseilinea sp.” showed a similar vertical distribution, *i.e.*, presence in deeper layers in summer than in winter, which may be attributed to the predicted deeper penetration of NIR light into the mats in summer (Fig. 5). The next relative of the least abundant chlorophototrophic *Chloroflexi*, ”*Ca.* Chloranaerofilum sp.” has been suggested to synthesize BChls *a* and *c* to capture light (64). In contrast to the reported strict anaerobic lifestyle of “*Ca.* Chloranaerofilum corporosum” in Mushroom Spring, YNP (62), the high relative abundance in the second layer of “*Ca.* Chloroanaerofilum sp.” in the summer sample (Fig. 5A) indicates tolerance to oxygen (Fig. 3). With only 98% nucleotide sequence identity compared to the proposed type species “*Ca.* Chloranaerofilum corporosum” (62), this phototroph recovered from Nakabusa hot springs appears to represent a new species of “*Ca.* Chloranaerofilum ”, with different metabolic traits, such as oxygen requirements or tolerance.

An additional four phototrophic bacteria were detected at a lower abundance and/or inconsistently (“*Ca.* Roseovibrio sp.” NK_OTU-032, *Elioraea* sp. NK_OTU-279 (*Proteobacteria*), “*Ca.* Thermochlorobacter sp.” (*Chlorobi*), and Chloracidobacterium sp. (*Acidobacteria*)) in the mats (Fig. S4, S5, and S6). The next relatives of the two phototrophic *Proteobacteria* in these mats, “*Ca.* Roseovibrio sp.” NK_ OTU-032 and *Elioraea* sp. NK_OTU-279, have been suggested to have similar growth patterns with respect to photosynthetic pigment compositions and tolerance to oxygen; both species contain BChl *a* and grow under aerobic conditions (62). Differences in the vertical distribution of the two members, as shown in Fig. 6, indicate slightly different ecological niches and potentially different preferences or needs for oxygen concentrations and/or light intensity. Nucleotide sequence identities of 98 and 94% between the sequences obtained in the present study and the proposed type strain species “*Ca.* Roseovibrio tepidum” strain MS-P3 (62) and “*Ca.* Elioraea thermophila” clone MS-B_OTU-4 (62), respectively, indicate different species and possibly even genera for the organisms in Nakabusa. The phototrophic *Chlorobi* member “*Ca.* Thermochlorobacter sp.” NK_OTU-021 represents a close relative to “*Ca.* Thermochlorobacter aerophilum”. The latter was initially detected in microbial mats from YNP using metagenomics as well as metatranscriptomic analyses (25, 32) and is the first aerobic photoheterotrophic member of the phylum *Chlorobi*, a phylum that is known for its strictly anaerobic sulfur-oxidizing chlorophototrophic members, the green sulfur bacteria (32). Despite being less frequently detected in the samples, the vertical distribution of “*Ca.* Thermochlorobacter sp.” NK_OTU-021 sequences in May 2017 revealed that the sequences of this chlorophototroph were the most abundant in the uppermost portion of the mat (Fig. 6A), in which the oxygenic phototrophic cyanobacteria, *Thermosynechococcus* spp., were the most abundant, thereby supporting the suggested need for high oxygen concentrations for this novel phototrophic member of *Chlorobi* (32, 62). Only a few sequences related to *Chloracidobacterium* sp. (NK_OTU-15047) were obtained in the present study. Based on the close similarity (97% nucleotide sequence identity) to the type strain *Cab. thermophilum*, a microaerophilic chlorophototrophic lifestyle may be assumed (60).

The vertical distribution of different chemotrophic members in the mats positively or negatively correlates with oxygen concentrations obtained in the present study (Fig. 3). For example, the abundant chemoheterotrophic “*Ca.* Thermonerobacter sp.” represented by NK_OTU-051 in the present study, increased in abundance with depth (Fig. 7), suggesting preferred growth in the anaerobic layers of the mats. It has recently been described as a putative anaerobe with dissimilatory sulfate-reducing metabolism by metagenomic and metatranscriptomic analyses (66). Therefore, its highest abundance in deeper, anaerobic layers in these mats is consistent with the general occurrence of sulfate reducers in anaerobic niches (7, 27, 39). In addition to the anaerobic respiration of sulfate, aerobic respiration in this bacterium was suggested from the findings of metagenomic analyses (66), which may be responsible for the detection of this organism in the microaerobic middle layer of the mat (Fig. 7). An increase in sequence abundance with depth indicating an anaerobic lifestyle was also observed for other abundant chemotrophic members of the mats, such as the uncultured ‘Chlorobi lineage 2’ (SM1HO2) member represented by NK_OTU-012 (Fig. 7). In contrast, uncultured ‘Chlorobi lineage 5’ NK_OTU-023 appears to tolerate oxygen in the middle to upper layers, suggesting an microaerobic or aerobic lifestyle. The uncultured *Armatimonadetes* member NK_OTU-028, similar to “Type OS-L” obtained from Mushroom and Octopus Springs in YNP, shows a vertical distribution indicating a possible microaerobic lifestyle, while previous studies suggested aerobic metabolism for the YNP mat member (Fig. 7) (59, 64). This indicates high metabolic versatility for different species, populations, or ecotypes, and correlates with predicted microdiversity in this taxonomic group, as suggested previously (63). Another abundant chemoheterotrophic member, the uncultured *Acidobacteria* member NK_OTU-007 was present in all samples, indicating a stable presence as a member of the core community in these mats. The vertical distribution of this mat member clearly revealed an indication for microaerobic to aerobic metabolism, which is further supported by the results of the metagenome analysis (Fig. 7, unpubl. data, Martinez *et al.*, in preparation).

## Conclusion

Green phototrophic microbial mats developing at approx. 60°C in Nakabusa hot springs (Nagano, Japan) consisted of a low diversity core community of nine abundant (≥1% mean relative sequence abundance) members, as well as approximately 28 low-abundance and variable community members in this 16S rRNA gene amplicon sequencing study. Ten different phototrophic bacteria were identified, representing five out of seven known phyla containing phototrophic members; three anoxygenic phototrophic *Chloroflexi* and one oxygenic *Cyanobacteria* species dominated the community. Oxygenic cyanobacteria were limited to the green upper 2-mm depth of the mat, as identified by 16S rRNA gene sequences and irradiance absorption spectra. Anoxygenic phototrophs were additionally found in the lower orange-colored layers, which correlated with the penetration of NIR light deeper into the mat. The vertical distribution of the different phototrophic bacteria indicates a number of ecological niches in part driven by micro-environmental gradients with regard to light and oxygen. This niche differentiation enables the co-existence of diverse chlorophototrophs in metabolically diverse communities in these mats. Furthermore, five abundant (≥1%) uncultured chemotrophic members with different lifestyles varying from predicted aerobic and microaerobic to anaerobic metabolism showed positive and negative correlations, respectively, with oxygen concentrations in their vertical distribution. Further studies on metabolic potential using metagenome sequence data (Martinez *et al.*, in preparation) will provide a better insight and further clarify the diversity and ecological potentials of the microbial mat community members in Nakabusa hot springs.

## SUPPLEMENTARY MATERIAL



## Figures and Tables

**Table 1:** Diversity indices based on sequence reads of OTUs from six sampling time points.

Sample Code	Date of Sampling	Chao1	CV (%)	OTUs*	*H*′	*H_max_*	E
GP_61	2016 June	24130	90.5	947	4.58	6.85	0.67
GP_64	2016 July	23161	90.6	868	4.86	6.77	0.72
GP_57	2016 July	20482	91.7	866	4.37	6.76	0.65
GP_56	2016 Nov	5952	98.8	708	3.69	6.56	0.56
GPL_56_M	2017 May	4421	99.1	859	4.02	6.76	0.60
GPL_56_N	2017 Nov	5613	98.8	452	3.22	6.11	0.53

**Mean**		**13960**	**94.9**	**783**	**4.12**	**6.64**	**0.62**
**SD**		**9544**	**4.4**	**180**	**0.60**	**0.27**	**0.07**

Chao1—Species richness estimation with singleton and doubleton sequences calculated by SpadeR (5)

CV—Coverage estimate of the entire dataset

OTUs*—number of OTUs without singleton

*H*′—Shannon Diversity Index

*H_max_* (maximum diversity of a sample) = In S (normal logarithm of S), where S is the total number of OTUs

E—Evenness = *H*′*/H_max_*

**Table 2:** Taxonomic affiliation and mean relative abundance of 37 selected microbial community members from six sampling time points based on 16S rRNA amplicon sequence reads. OTU selection was based on one of the sampling time points (November 2016) with ≥0.5% relative sequence abundance. Identities were based on nucleotide sequence similarities with their closest relatives in NCBI databases using BLAST hits.

Phylum	OTU-name	Mean Relative Abundance	Min	Max	SD	Relevant BLAST Hits	Acc. No.	Identity (% nt)
*Chloroflexi*	NK_OTU-002^P^	19.5%	5.21%	38.20%	12.31%	clone NLEA-OTU2 (Nakabusa hot springs, Japan)	MF435938	100
						*Roseiflexus castenholzii* HLO8^T^	NR_112114	100
	NK_OTU-001^P^	17.6%	2.64%	31.09%	9.34%	clone NKB_63_10 (Nakabusa hot springs, Japan)	JF826984	100
						*Chloroflexus aggregans* DSM 9485^T^	NR_074226	99
	NK_OTU-006^P^	1.8%	1.04%	2.94%	0.92%	clone NLEA-OTU120 (Nakabusa hot springs, Japan)	MF435983	100
						“*Candidatus* Roseilinea gracile” (Mushroom Spring, YNP, USA)	KY937207	96
						*Leptolinea tardivitalis* YMTK-2^T^	NR_040971	88
	NK_OTU-014	0.3%	0.10%	0.88%	0.30%	clone NLEA-OTU35 (Nakabusa hot springs, Japan)	MF435955	100
						clone iTag MS-B_2012_OTU-9 (Mushroom Spring, YNP, USA)	KU860149	99
						*Thermomarinilinea lacunifontana* SW7^T^	NR_132293	92
	NK_OTU-222^P^	0.5%	0.0%	2.85%	1.16%	clone NLEA-OTU27 (Nakabusa hot springs, Japan)	MF435959	100
						“*Candidatus* Chloranaerofilum corporosum” (Mushroom Spring, YNP, USA)	KY937209	98
						*Oscillochloris trichoides* DG-6^T^	AF146832	92
	NK_OTU-092^P^	0.2%	0.0%	1.08%	0.43%	*Chloroflexus* sp. clone Alla12-1 (Alla hot spring, Russia)	KP701483	100
						*Chloroflexus aurantiacus* J-10-fl T	NR_074263	100

*Cyanobacteria*	NK_OTU-003^P^	6.5%	3.35%	10.74%	2.69%	*Thermosynechococcus* sp. NK55 (Nakabusa hot springs, Japan)	CP006735	100
						*Thermosynechococcus elongatus* PKUAC-SCTE731	MF405428	100

*Acidobacteria*	NK_OTU-007	5.9%	0.18%	13.71%	5.69%	uncultured denovo34541 (Nakabusa hot springs, Japan)	LC381388	100
						*Acidobacteria* clone YNP_SBC_BP4_B26 (Lower Geyser Basin, YNP, USA)	HM448257	96
						*Chloracidobacterium thermophilum* D	KP300942	87
	NK_OTU-046	0.6%	0.25%	1.47%	0.47%	clone NLEA-OTU13 (Nakabusa hot springs, Japan)	MF435947	100
						*Acidobacteria* clone Tyva_DA_OTU_0059 (Hydrothermal spring, Russia)	MG950134	100
						*Paludibaculum fermentans* P105^T^	NR_134120	95
	NK_OTU-15047^P^	0.001%	0.0%	0.01%	0.003%clone iTag MS-B_2012_OTU-17 (Mushroom Spring, YNP, USA)		KU860157	100
						*Chloracidobacterium thermophilum* D	KP300942	97

*Armatimonadetes*	NK_OTU-028	4.3%	0.08%	14.28%	5.84%	clone NKB_56_N2 (Nakabusa hot springs, Japan)	JF826973	100
						clone iTag MS-B_2012_OTU-3 (Mushroom Spring, YNP, USA)	KU860143	98
						*Eubacterium* sp. (OS type L) (Octopus Spring, YNP, USA)	L04707	96

*Chlorobi/Bacteroidetes/Ignavibacteria*	NK_OTU-012	1.7%	0.07%	4.89%	1.67%	clone NKB_56_U2 (Nakabusa hot springs, Japan)	JF826976	100
						*Chlorobi* clone SM1H02 (Mammoth hot springs, YNP, USA)	AF445702	99
						*Ignavibacterium album* JCM16511^T^	NR_074698	89
	NK_OTU-023	1.7%	0.59%	5.77%	2.05%	clone iTag MS-B_2012_OTU-24 (Mushroom Spring, YNP, USA)	KU860164	100
						Uncultured *Rhodothermus* sp. clone 9 (Porcelana hot spring, Chile)	MH938161	99
						“*Candidatus* Rhodothermus clarus”	AB252420	85
	NK_OTU-051	1.0%	0.08%	3.35%	1.08%	*Chlorobi* clone: HGM-D-87 (Geothermal water, Kagoshima, Japan)	AB539665	99
						clone iTag MS-B_2012_OTU-29 (Mushroom Spring, YNP, USA)	KU860169	99
						*Ignavibacterium album* JCM16511^T^	NR_074698	81
	NK_OTU-008	0.8%	0.01%	2.04%	0.81%	*Bacteroidetes* clone Tyva_DA_OTU_0019 (Hydrothermal spring, Russia)	MG950110	100
						*Ignavibacterium album* JCM16511^T^	NR_074698	95
	NK_OTU-259	1.0%	0.0%	5.77%	2.35%	clone NLEA-OTU41 (Nakabusa hot springs, Japan)	MF435972	100
						clone SJA-28 (Germany)	AJ009458	88
						*Thauera mechernichensis* TL1^T^	NR_026473	87
	NK_OTU-021^P^	0.1%	0.0%	0.62%	0.25%	*Chlorobium* sp. clone 4 (Porcelana hot spring, Chile)	MH938157	99
						clone OS-GSB (Octopus Spring, YNP, USA)	KU565869	91
						*Chloroherpeton thalassium* ATCC 35110^T^	NR_074270	92
	NK_OTU-005	2.9%	0.0%	14.09%	5.62%	*Bacteroidetes* clone A5-00YK9 (Boekleung hot spring, Thailand)	EU376411	99
						Candidate division OP clone M2UF07 (YNP, USA)	FJ885732	95
						*Lewinella maritima* HME9321^T^	NR_158053	89
	NK_OTU-004	1.2%	0.0%	4.90%	2.01%	clone NLEA-OTU36 (Nakabusa hot springs, Japan)	MF435960	99
						clone iTag MS-B_2012_OTU-196 (Mushroom Spring, YNP, USA)	KU860334	92
						*Pedobacter steynii* DSM19110^T^	MH929833	84
	NK_OTU-016	0.2%	0.0%	0.84%	0.33%	*Bacteroidetes* clone YNP_SBC_MS3_B92 (Lower Geyser Basin, YNP, USA)	HM448200	97
						clone Tat-08-003_12_90 (El Tatio Geyser Field, Chile)	GU437354	96
						*Solitalea* longa HR-AV^T^	MF685247	89

*Aquificae*	NK_OTU-010	0.9%	0.19%	2.33%	0.80%	uncultured denovo14971 (Nakabusa hot springs, Japan)	LC381396	100
						*Sulfurihydrogenibium azorense* Az-Fu1^T^	NR_102858	100
						*Sulfurihydrogenibium yellowstonense* SS-5^T^	NR_043111	94
	NK_OTU-019	0.4%	0.08%	0.76%	0.28%	uncultured denovo29919 (Nakabusa hot springs, Japan)	LC381395	100
						clone dongzy2tff41747 (Tibet hot spring, China)	KU482385	97
						*Thermocrinis jamiesonii* GBS1^T^	NR_145905	96
	NK_OTU-015	0.4%	0.06%	0.88%	0.32%	*Hydrogenobacter* sp. clone Tsenher12otu8-10 (Tsenher hot spring, Mongolia)	KT258797	100
						uncultured denovo15700 (Nakabusa hot springs, Japan)	LC381391	100
						*Hydrogenobacter subterraneus* HGP1^T^	NR_024729	99

*Planctomycetes*	NK_OTU-009	0.9%	0.17%	2.10%	0.83%	Uncultured *Eubacterium* env. OPS 3 (Obsidian Pool, YNP, USA)	AF018188	100
						“*Candidatus* Gemmata massiliana” IIL29	NR_148576	95
	NK_OTU-033	0.4%	0.12%	1.10%	0.38%	clone TP19 (Tibet hot spring, China)	EF205574	99
						clone iTag MS-B_2012_OTU-51 (Mushroom Spring, YNP, USA)	KU860191	98
						*Thermogutta terrifontis* R1^T^	NR_134826	90

*Thermodesulfobacteria*	NK_OTU-013	0.3%	0.003%	0.97%	0.44%	uncultured denovo155 (Nakabusa hot springs, Japan)	LC381408	100
						*Caldimicrobium thiodismutans* TF1^T^	NR_148865	100
						*Caldimicrobium rimae* DS^T^	NR_044283	97

*Proteobacteria*	NK_OTU-030	0.4%	0.10%	0.69%	0.22%	clone Alla11otu10-1 (Alla hot spring, Russia)	KP676764	99
						clone NLEA-OTU15 (Nakabusa hot springs, Japan)	MF435964	99
						*Thermomonas hydrothermalis* SGM-6^T^	NR_025265	92
	NK_OTU-032^P^	0.6%	0.43%	0.99%	0.22%	clone B35 (Great Artesian Basin, Australia)	AF407720	100
						clone NLEA-OTU29 (Nakabusa hot springs, Japan)	MF435979	99
						“*Candidatus* Roseovibrio tepidum” MS-P3	MG821467	98
	NK_OTU-031	1.2%	0.0%	3.61%	1.64%	clone iTag MS-B_2012_OTU-92 (Mushroom Spring, YNP, USA)	KU860232	100
						clone QL15B_6pJ (Queen’s Laundry hot spring, YNP, USA)	KU382142	100
						“*Candidatus* Desulfacinum subterraneum”	AF385080	90
	NK_OTU-017	0.1%	0.0%	0.81%	0.33%	*Desulfomicrobium* sp. 21	KX018622	100
						*Desulfomicrobium thermophilum* DSM 16697^T^	MH741285	99
	NK_OTU-279^P^	0.02%	0.0%	0.11%	0.04%	*Elioraea* sp. clone 5 (Porcelana hot spring, Chile)	MH938158	100
						*Elioraea tepidiphila* TU-7^T^	NR_044259	100
						“*Candidatus* Elioraea thermophila” MS-B_OTU-4	MH555907	94

*Deinococcus-Thermus*	NK_OTU-025	0.5%	0.10%	0.94%	0.27%	clone NKB_56_02 (Nakabusa hot Springs, Japan)	JF826974	100
						*Meiothermus luteus* YIM 72257^T^	NR_157749	99
*Firmicutes*	NK_OTU-018	0.4%	0.11%	0.79%	0.24%	uncultured denovo15330 (Nakabusa hot Springs, Japan)	LC381401	100
						*Firmicutes* clone YNP_SBC_BP3_B7	HM448232	95
						*Thermodesulfitimonas autotrophica* SF97^T^	NR_156074	87

*Spirochaetae*	NK_OTU-011	0.3%	0.07%	1.18%	0.43%	clone iTag MS-B_2012_OTU-25 (Mushroom Spring, YNP, USA)	KU860165	100
						clone NKB48 (Nakabusa hot springs, Japan)	FR691784	100
						*Leptonema illini* 3055^T^	NR_043139	86

*Nitrospirae*	NK_OTU-020	0.9%	0.08%	2.19%	0.78%	clone NLEA-OTU9 (Nakabusa hot springs, Japan)	MF435944	100
						clone NKB_63_50 (Nakabusa hot springs, Japan)	JF826987	100
						*Thermodesulfovibrio yellowstonii* DSM 11347^T^	NR_074345	94
	NK_OTU-514	0.2%	0.0%	1.11%	0.45%	clone G19 (Great Artesian Basin, Australia)	AF407702	100
						“*Candidatus* Nitrospira calida” (Geothermal spring, Austria)	HM485589	96

*Parcubacteria*	NK_OTU-034	0.2%	0.05%	0.46%	0.17%	clone HGM-U-39 (Geothermal water, Kagoshima, Japan)	AB539626	96
						clone iTag MS-B_2012_OTU-222 (Mushroom Spring, YNP, USA)	KU860360	88
						*Parcubacteria* group bacterium GW2011_GWC1_41_7	KX123526	79

highlighted—Core members of the community (OTU was detected in all samples and with relative abundance of ≥ 0.003% in each of the sampling time points)

P —phototrophic member based on its closest relative

T —type strain

nt—nucleotide sequence similarity

0.0%—relative abundance reads of <0.003% or zero sequence reads
