# Supplementary material for: Vertical Distribution and Diversity of Phototrophic Bacteria within a Hot Spring Microbial Mat (Nakabusa Hot Springs, Japan)
Source: Microbes Environ. 2019 Dec 27;34(4):374–87. doi: 10.1264/jsme2.ME19047 (PMC6934398; doi:10.1264/jsme2.ME19047)
Supplement: Supplementary file 1 [file 34_374_s1.pdf]

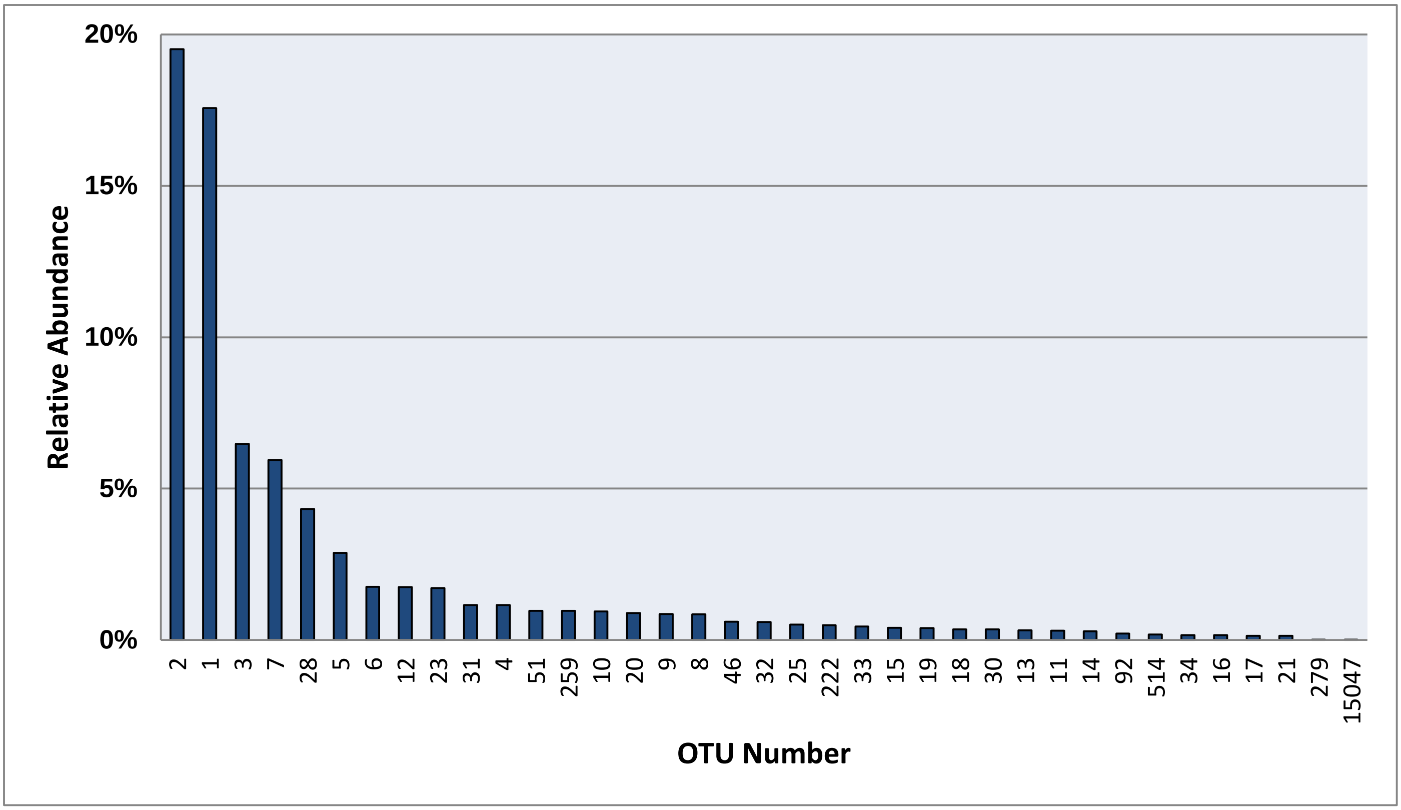

Fig. S1. Rank abundance plot of the selected 37 OTUs based on the mean relative abundance of the 16S rRNA gene amplicon sequences obtained from the six sampling time points in this study.

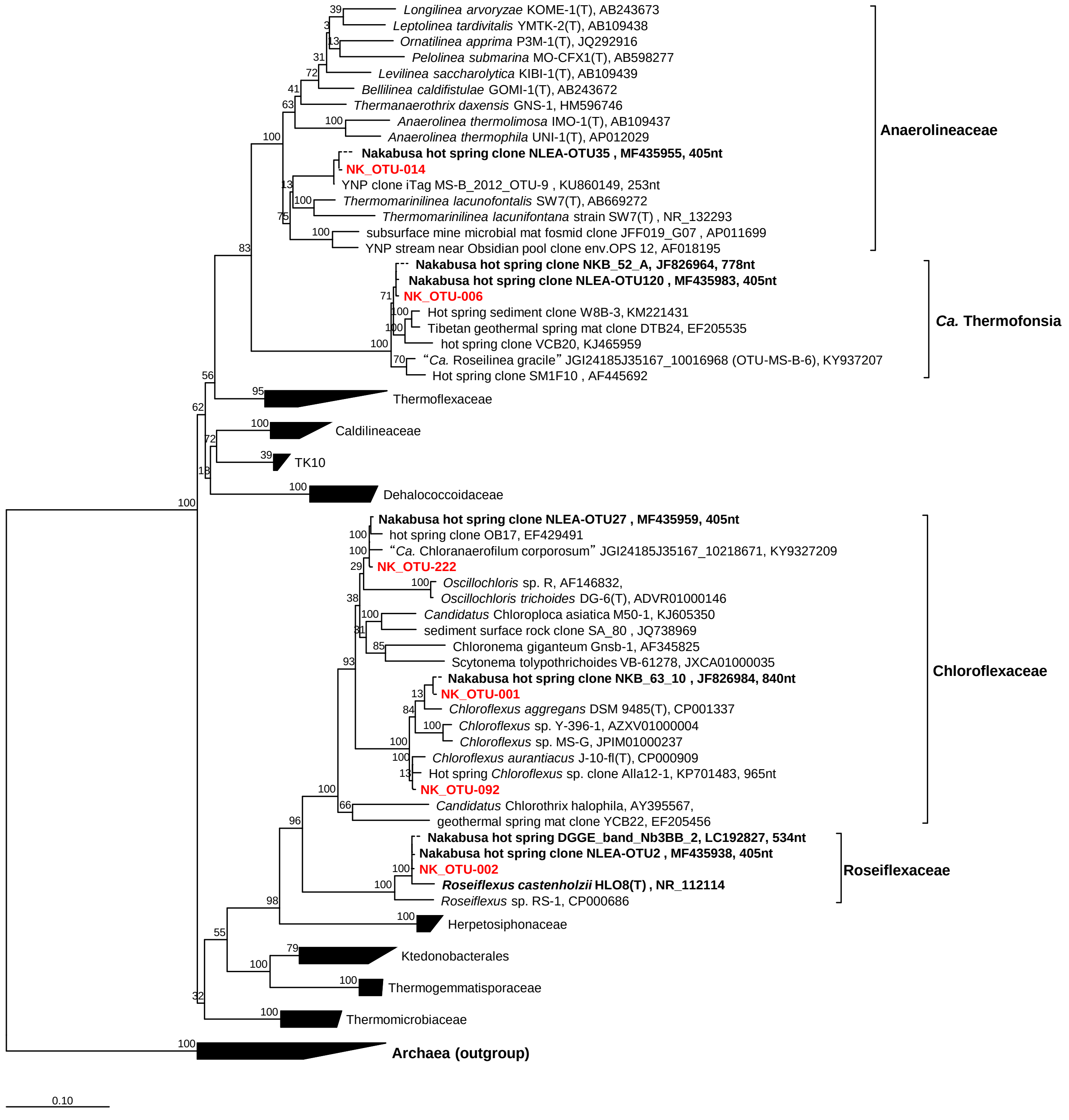

Fig. S2. Phylogenetic tree based on the 16S rRNA sequences showing the phylogenetic relationship of the OTUs obtained in this study and sequences obtained from other hot springs belonging to phylum *Chloroflexi*. OTU numbers in this study are shown in red bold (253 bp sequence length) while other sequences from Nakabusa hot springs are shown in black bold. Only sequences with length >1000 bp were used for phylogenetic calculations. Sequence length <1,000 bp were added using the Parsimony method without changing tree topology.

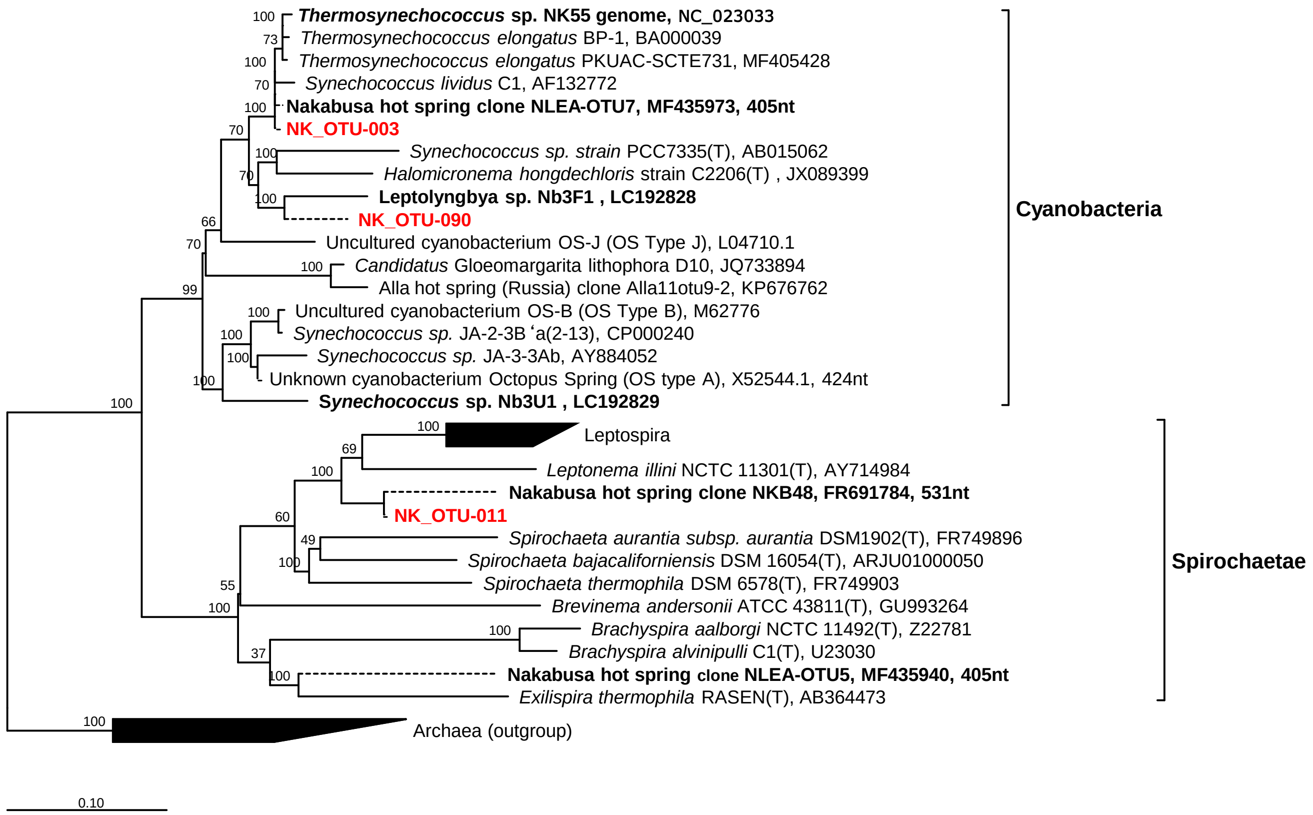

Fig. S3. Phylogenetic tree based on the 16S rRNA sequences showing the phylogenetic relationship of the OTUs obtained in this study and sequences obtained from other hot springs belonging to phyla *Cyanobacteria* and *Spirochaete.* OTU numbers in this study are shown in red bold (253 bp sequence length) while other sequences from Nakabusa hot springs are shown in black bold. Only sequences with length >1000 bp were used for phylogenetic calculations. Sequence length <1,000 bp were added using the Parsimony method without changing tree topology.

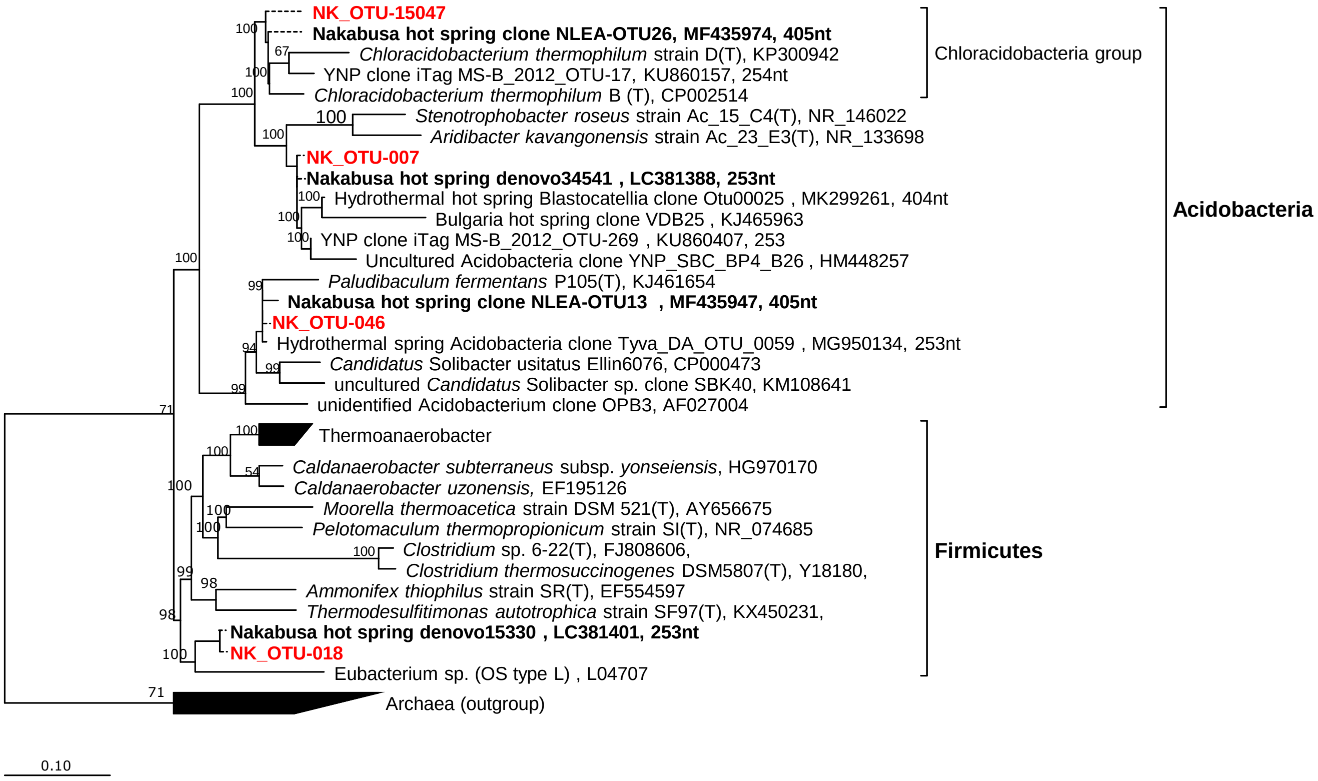

Fig. S4. Phylogenetic tree based on the 16S rRNA sequences showing the phylogenetic relationship of the OTUs obtained in this study and sequences obtained from other hot springs belonging to phyla *Acidobacteria* and *Firmicutes.* OTU numbers in this study are shown in red bold (253 bp sequence length) while other sequences from Nakabusa hot springs are shown in black bold. Only sequences with length >1000 bp were used for phylogenetic calculations. Sequence length <1,000 bp were added using the Parsimony method without changing tree topology.

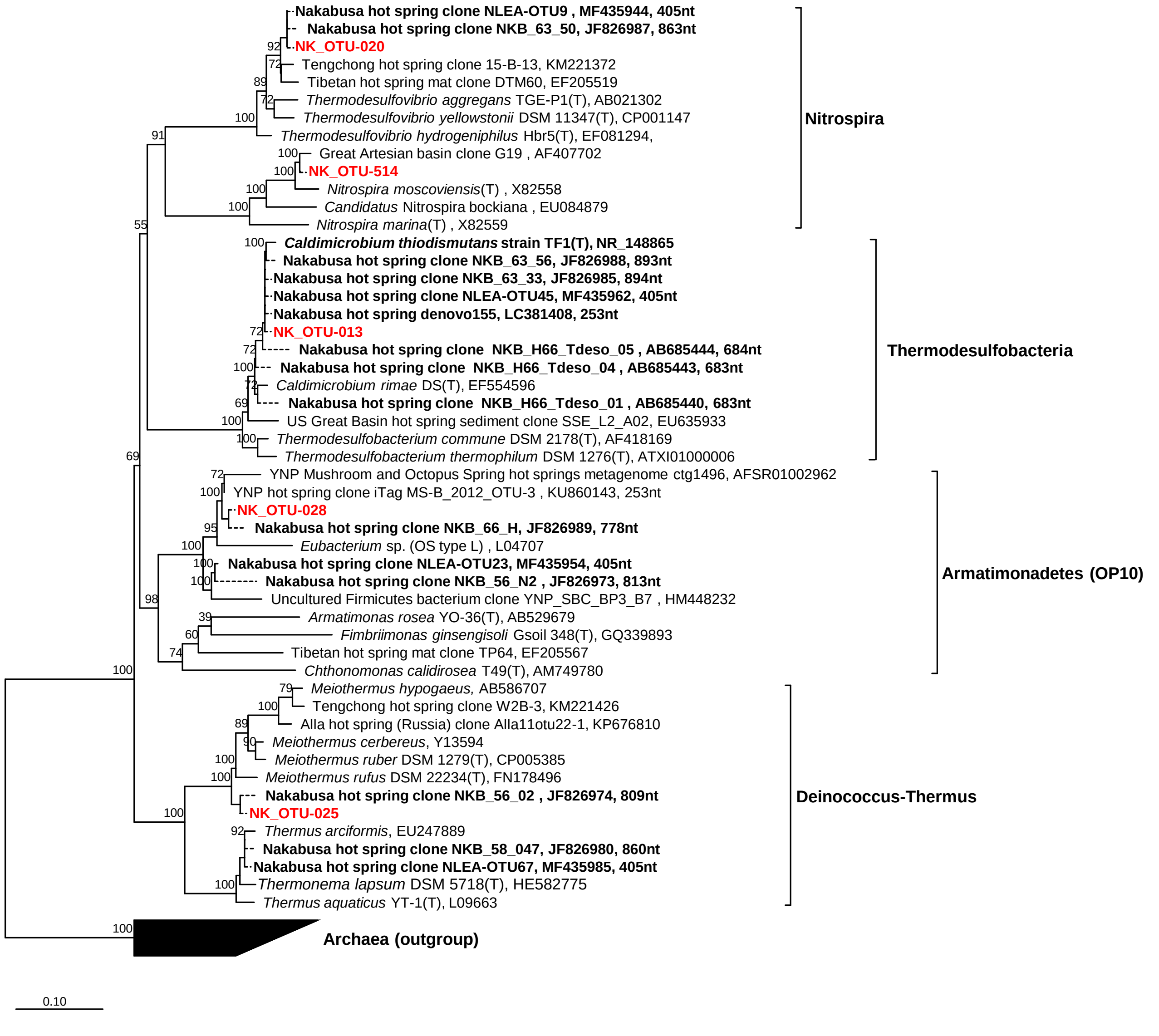

Fig. S5. Phylogenetic tree based on the 16S rRNA sequences showing the phylogenetic relationship of the OTUs obtained in this study and sequences obtained from other hot springs belonging to phyla *Armatimonadetes, Deinococcus-Thermi, Nitrospira and Thermodesulfobacteria.* OTU numbers in this study are shown in red bold (253 bp sequence length) while other sequences from Nakabusa hot springs are shown in black bold. Only sequences with length >1000 bp were used for phylogenetic calculations. Sequence length <1,000 bp were added using the Parsimony method without changing tree topology.

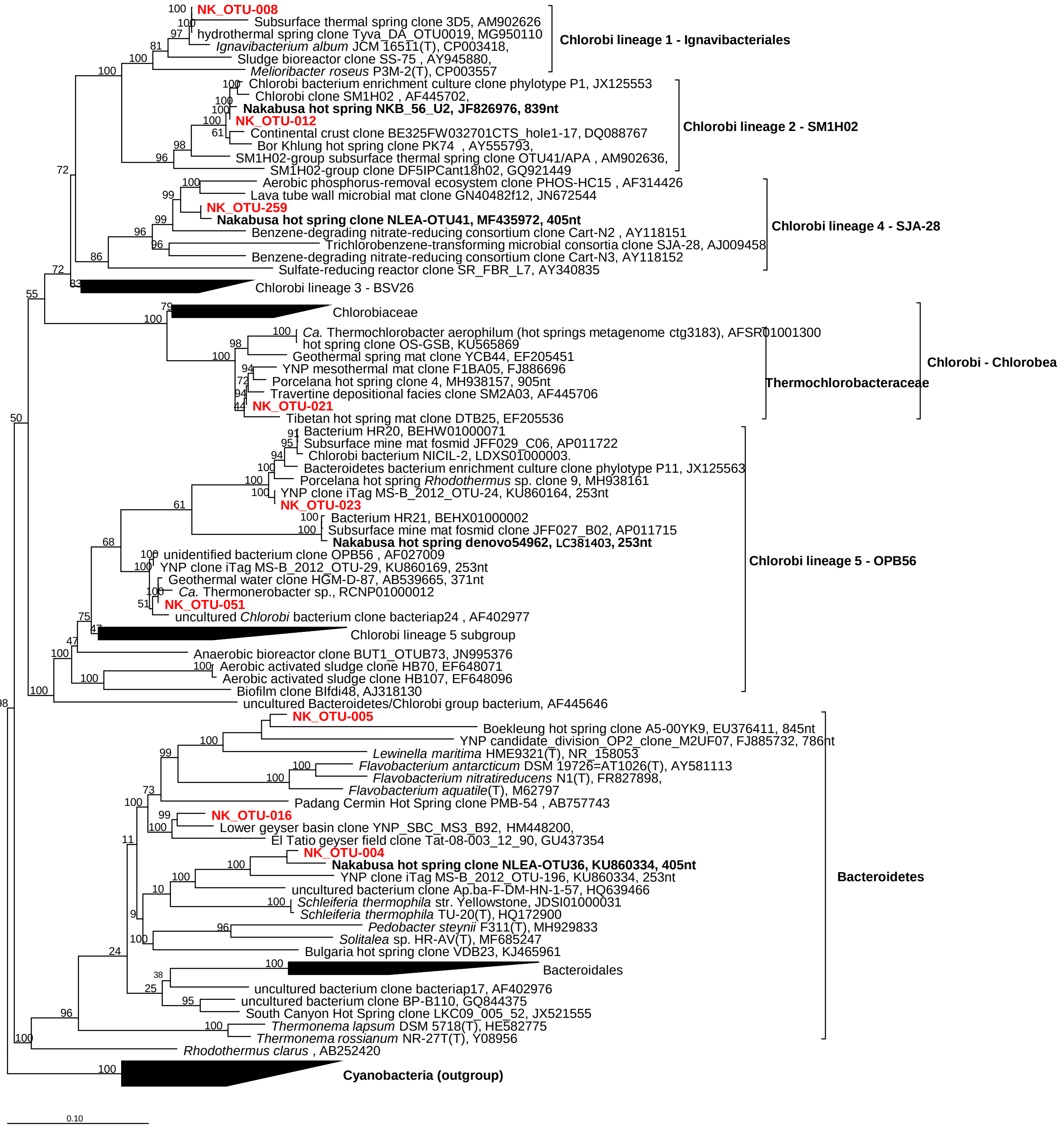

Fig. S6. Phylogenetic tree based on the 16S rRNA sequences showing the phylogenetic relationship of the nine OTUs obtained in this study and sequences obtained from other hot springs belonging to *Chlorobi/Bacteroidetes/Ignavibacteria* superphylum. OTU numbers in this study are shown in red bold (253 bp sequence length) while other sequences from Nakabusa hot springs are shown in black bold. Only sequences with length >1000 bp were used for phylogenetic calculations. Sequence length <1,000 bp were added using the Parsimony method without changing tree topology.

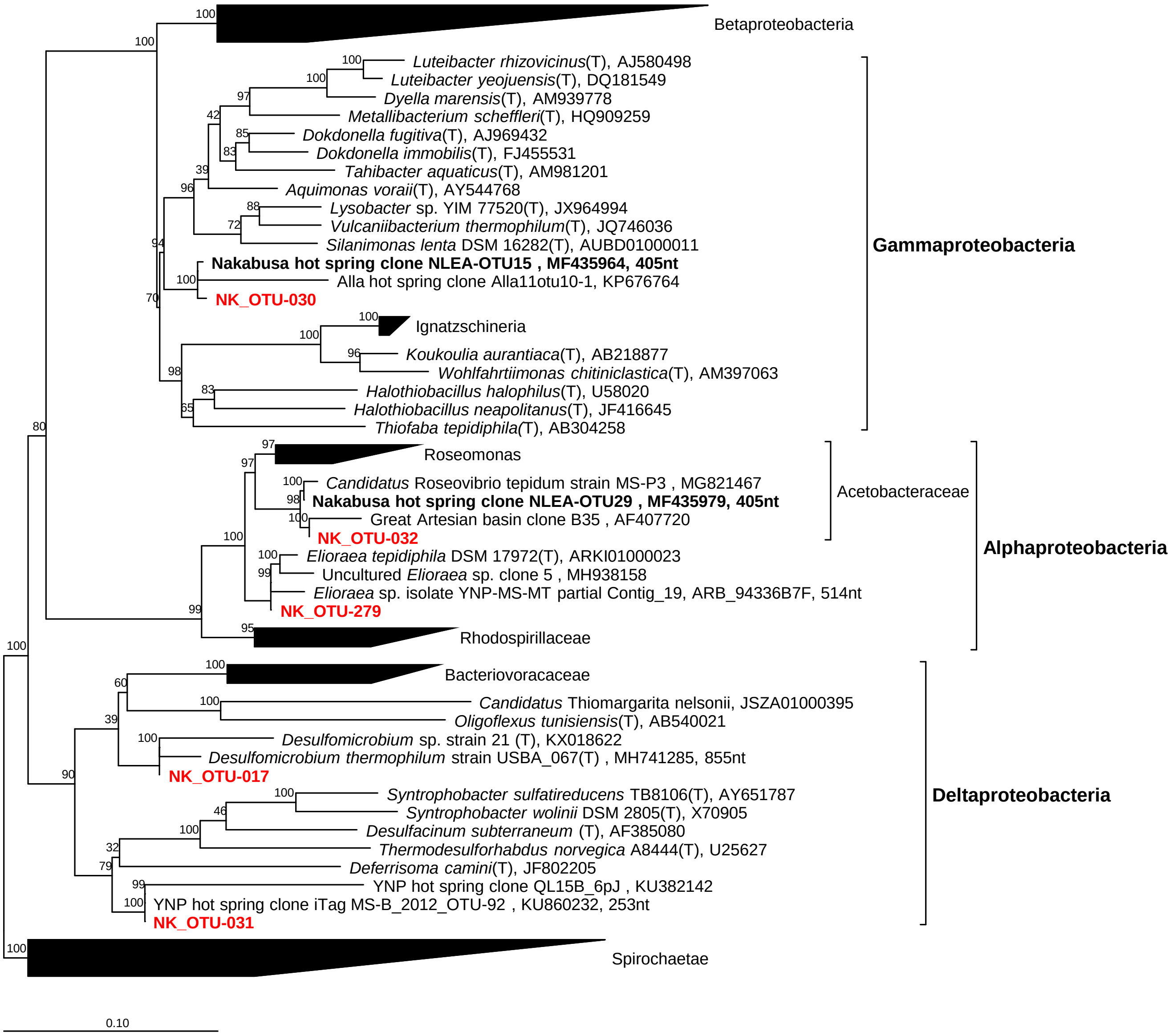

Fig. S7. Phylogenetic tree based on the 16S rRNA sequences showing the phylogenetic relationship of the OTUs obtained in this study and sequences obtained from other hot springs belonging to phylum *Proteobacteria.* OTU numbers in this study are shown in red bold (253 bp sequence length) while other sequences from Nakabusa hot springs are shown in black bold. Only sequences with length >1000 bp were used for phylogenetic calculations. Sequence length <1,000 bp were added using the Parsimony method without changing tree topology.

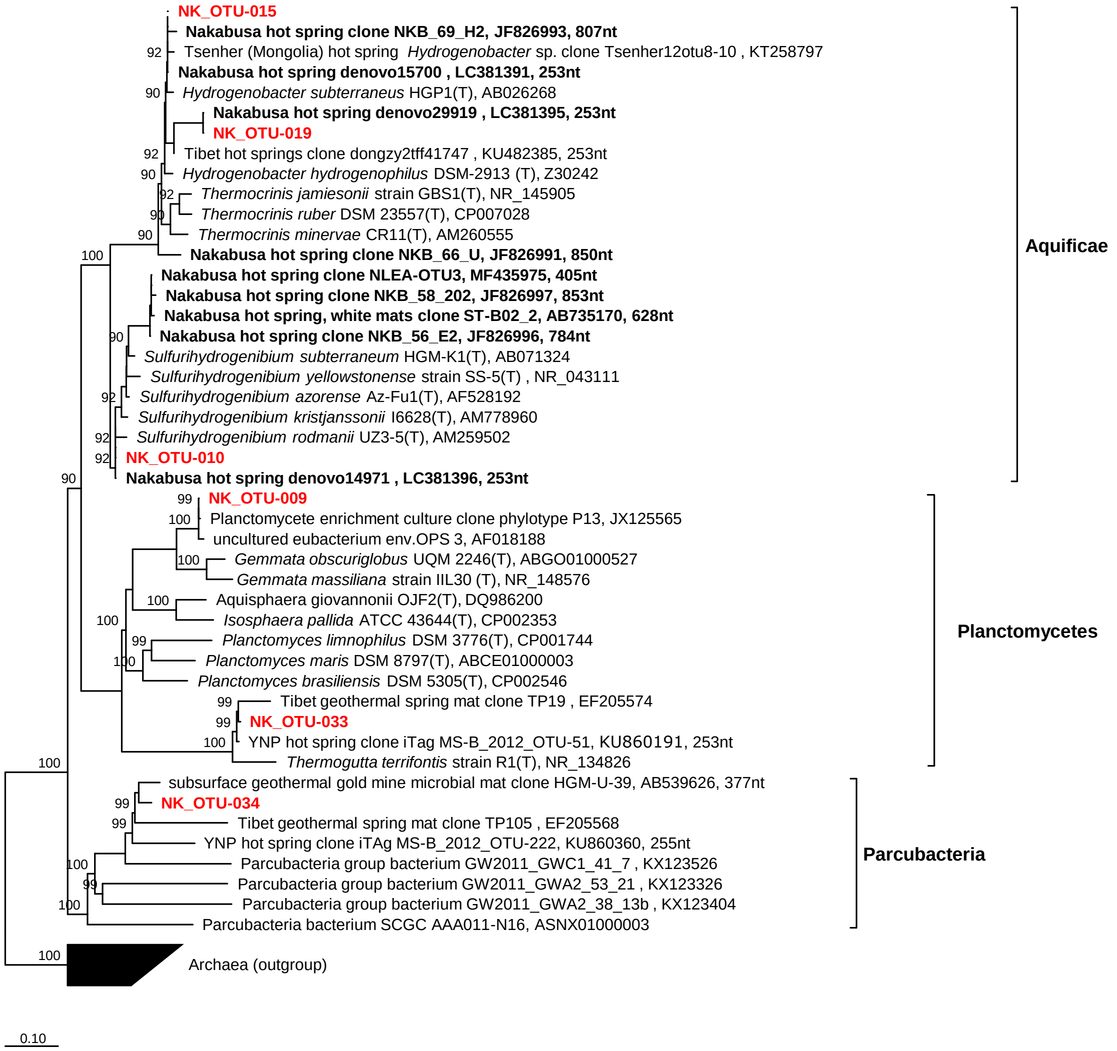

Fig. S8. Phylogenetic tree based on the 16S rRNA sequences showing the phylogenetic relationship of the OTUs obtained in this study and sequences obtained from other hot springs belonging to phyla *Aquificae, Planctomycetes* and *Parcubacteria.* OTU numbers in this study are shown in red bold (253 bp sequence length) while other sequences from Nakabusa hot springs are shown in black bold. Only sequences with length >1000 bp were used for phylogenetic calculations. Sequence length <1,000 bp were added using the Parsimony method without changing tree topology.

Table S1. Description of samples collected from Nakabusa hot spring 'Stream Site= Site B' (Nagano Pref., Japan).

Sample Code	Description	Sampling point	Water Temp (°C)	Date of Sampling	Method of DNA Extraction
GP_61	Upper green and orange undermat, core samples	Green Pool	61	Jun-16	CTAB method[A]
GP_64	Upper green and orange undermat, core samples	Green Pool	64	Jul-16	CTAB method[A]
GP_57	Upper green and orange undermat, core samples	Green pool	57	Jul-16	CTAB method[A]
GP_56	Upper green and orange undermat, core samples	Green pool	56	Nov-16	MO BIO PowerBiofilm Kit[B]
GPL_56_M	Five separated layers, falcon tube samples	Green Pool	56	May-17	MO BIO PowerBiofilm Kit[B]
GPL_56_N	Five separated layers, falcon tube samples	Green Pool	56	Nov-17	MO BIO PowerBiofilm Kit[B]

[A] – Cetyl trimethylammonium bromide (CTAB) method (Nishihara et al., 2018)

[B] – MO BIO PowerBiofilm DNA Isolation Kit (QIAGEN Inc., USA)

Table S2. Number of sequence reads recovered from 16S rRNA amplicon analyses from six sampling time-points.

Sample Code	Number of Samples	Total Number of Reads with Singleton	Total Number of Reads without Singleton	Number of OTUs with Singleton	Number of OTUs* without Singleton	Singleton Sequences
GP_61	1 mat core	40415	36562	4800	947	3853
GP_64	1 mat core	40154	36378	4644	868	3776
GP_57	1 mat core	41491	38050	4307	866	3441
GP_56	6 mat cores	109174	105425	1849	708	1141
GPL_56_M	5 mat layers	125619	119913	1745	859	886
GPL_56_N	5 mat layers	73729	72842	1345	452	893

*used in the calculation of relative abundance

Table S3. Comparison of the differences of the mean relative abundances of vertical distribution of phototrophs between May and November 2017 using T-test as statistical tool.

OTU-Number	Identity	2017 May							2017 November							T-test (p-value)
		Relative Abundance in Vertical Layers					Mean	SD	Relative Abundance in Vertical Layers					Mean	SD	
		L5	L4	L3	L2	L1			L5	L4	L3	L2	L1			
NK_OTU-003	*Thermosynechococcus sp.*	1.035%	1.315%	2.950%	12.375%	20.097%	7.555%	0.0841	2.917%	1.073%	0.588%	3.212%	20.338%	5.626%	0.0830	0.72
NK_OTU-002	*Roseiflexus castenholzii*	25.456%	45.916%	37.961%	11.093%	7.891%	25.663%	0.1650	18.381%	11.512%	41.956%	71.366%	47.078%	38.059%	0.2397	0.37
NK_OTU-222	*Ca.* Chloranaerofilum sp.	0.030%	0.511%	5.119%	6.657%	2.227%	2.909%	0.0289	0.002%	0.026%	0.005%	0.046%	0.0%	0.016%	0.0002	0.09
NK_OTU-001	*Chloroflexus aggregans*	0.331%	1.078%	0.592%	5.833%	5.913%	2.750%	0.0286	24.801%	17.016%	29.748%	7.638%	12.011%	18.243%	0.0906	0.02*
NK_OTU-006	*Ca.* Roseilinea sp.	0.631%	1.856%	2.758%	1.651%	1.506%	1.681%	0.0076	0.040%	0.011%	0.538%	0.738%	1.753%	0.616%	0.0071	0.05*
NK_OTU-092	*Chloroflexus aurantiacus*	0.021%	0.050%	0.195%	2.161%	3.436%	1.173%	0.0155	0.011%	0.006%	0.010%	0.322%	0.206%	0.111%	0.0015	0.20
NK_OTU-032	*Ca.* Roseovibrio sp.	0.028%	0.135%	0.374%	1.228%	0.464%	0.446%	0.0047	0.336%	0.288%	0.496%	0.906%	0.643%	0.534%	0.0025	0.72
NK_OTU-279	*Elioraea sp.*	0.071%	0.107%	0.217%	0.061%	0.076%	0.106%	0.0006	0.0%	0.008%	0.015%	0.024%	0.0%	0.009%	0.0001	0.03*
NK_OTU-15047	*Chloracidobacterium sp.*	0.0%	0.0%	0.0%	0.0%	0.0%	0.0%	0.0000	0.017%	0.002%	0.007%	0.0%	0.017%	0.008%	0.0001	0.08
NK_OTU-021	*Ca.* Thermochlorobacter sp.	0.035%	0.043%	0.141%	0.478%	0.674%	0.274%	0.0029	0.0%	0.0%	0.0%	0.0%	0.0%	0.0%	0.0000	0.10

L5- bottom layer

L1- surface layer

*- there is a significant difference between sampling time points ($p \geq 0.05$)

0.0% - zero sequence reads

Table S4. Comparison of the differences of the mean relative abundances of vertical distribution of phototrophs between May and November 2017 using T-test as statistical tool. .

OTU-Number	Identity	2017 May							2017 November							T-test (p-value)
		Relative Abundance in Vertical Layers					Mean	SD	Relative Abundance in Vertical Layers					Mean	SD	
		L5	L4	L3	L2	L1			L5	L4	L3	L2	L1			
NK_OTU-012	*unc Chlorobi* lineage 2 (SM1H02)	2.446%	1.132%	0.069%	0.006%	0.001%	0.731%	1.072%	4.665%	4.226%	0.251%	0.005%	0.002%	1.830%	2.395%	0.39
NK_OTU-051	*Ca.* Thermonerobacter sp.	0.163%	0.146%	0.028%	0.002%	0.000%	0.068%	0.080%	1.041%	2.396%	0.636%	0.096%	0.003%	0.834%	0.969%	0.15
NK_OTU-023	*unc Chlorobi* lineage 5 (OPB56)	0.125%	0.067%	0.023%	0.165%	0.146%	0.106%	0.059%	0.500%	0.100%	1.200%	0.400%	0.800%	0.600%	0.418%	0.06
NK_OTU-007	*unc Acidobacteria (Subgroup4)*	0.165%	0.030%	0.024%	0.127%	0.693%	0.208%	0.278%	0.178%	0.032%	1.124%	0.895%	1.907%	0.827%	0.760%	0.15
NK_OTU-028	*unc Armatimonadetes (OS-L)*	0.820%	1.026%	0.308%	0.102%	0.113%	0.473%	0.424%	0.070%	0.031%	0.165%	0.071%	0.018%	0.071%	0.058%	0.10

L5- bottom layer

L1- surface layer

p≥0.05 - no significnat differences between sampling time points
